# Promoter editing of *LpNOL* generates stay-green perennial ryegrass with improved forage quality and heat tolerance

**DOI:** 10.1093/plphys/kiaf402

**Published:** 2025-09-30

**Authors:** Huadong Yang, Jiaming Yao, Huanhuan Hao, Li Jiang, Jieqin Li, Yingjun Chi, Jing Zhang, Bin Xu

**Affiliations:** College of Agro-Grassland Science, Nanjing Agricultural University, Nanjing 210095, People's Republic of China; College of Agro-Grassland Science, Nanjing Agricultural University, Nanjing 210095, People's Republic of China; College of Agro-Grassland Science, Nanjing Agricultural University, Nanjing 210095, People's Republic of China; State Key Laboratory of Desert and Oasis Ecology, Xinjiang Institute of Ecology and Geography, Chinese Academy of Sciences, Urumqi 830011, China; College of Agriculture, Anhui Science and Technology University, Fengyang 239000, Anhui, China; Anhui Province International Joint Research Center of Forage Bio-Breeding, Fengyang 239000, China; College of Agro-Grassland Science, Nanjing Agricultural University, Nanjing 210095, People's Republic of China; College of Agro-Grassland Science, Nanjing Agricultural University, Nanjing 210095, People's Republic of China; College of Agro-Grassland Science, Nanjing Agricultural University, Nanjing 210095, People's Republic of China

## Abstract

Editing the heat-responsive motif of the promoter of a chlorophyll *b* reductase gene, *NON-YELLOW COLORING 1-like* (*NOL*) generated stay-green perennial ryegrass mutants with improved forage quality and heat tolerance.

Dear Editor,

Perennial ryegrass (*Lolium perenne* L., abbreviated as PR) is the most widely grown forage and turf grass species in the world. One feature of PR is its fast leaf growth and senescence rates sustaining only 3.5 leaves in each tiller which constrains its biomass accumulation and forage quality improvement. The functional stay-green trait is particularly desirable for PR and economically important for all forage grasses. Here, we developed functional stay-green mutant PR by editing the promoter of *NON-YELLOW COLORING 1-like* (*NOL*).

NON-YELLOW COLORING 1 and NOL are a pair of chlorophyll (Chl) *b* reductases catabolizing Chl*b* (Sato et al. 2009). Constitutive RNA interference of *LpNOL* (*LpNOL*-RNAi, abbreviated as *NOLi*) led to significantly more green leaves (Yu et al. 2021), improved heat tolerance (Yu et al. 2022), but reduced tillering in PR. The expression of *LpNOL* was heat-inducible (Yu *et al.* 2022). The degree of heat-induced Chl loss was significantly correlated with the expression levels of *LpNOL* in 12 PR varieties with different heat tolerance (Fig. 1, A to D). We hypothesized that reducing heat-inducible expression levels of *LpNOL* could increase PR's heat tolerance with little or no vegetative growth penalty (e.g. tillering capacity).

**Figure 1.**
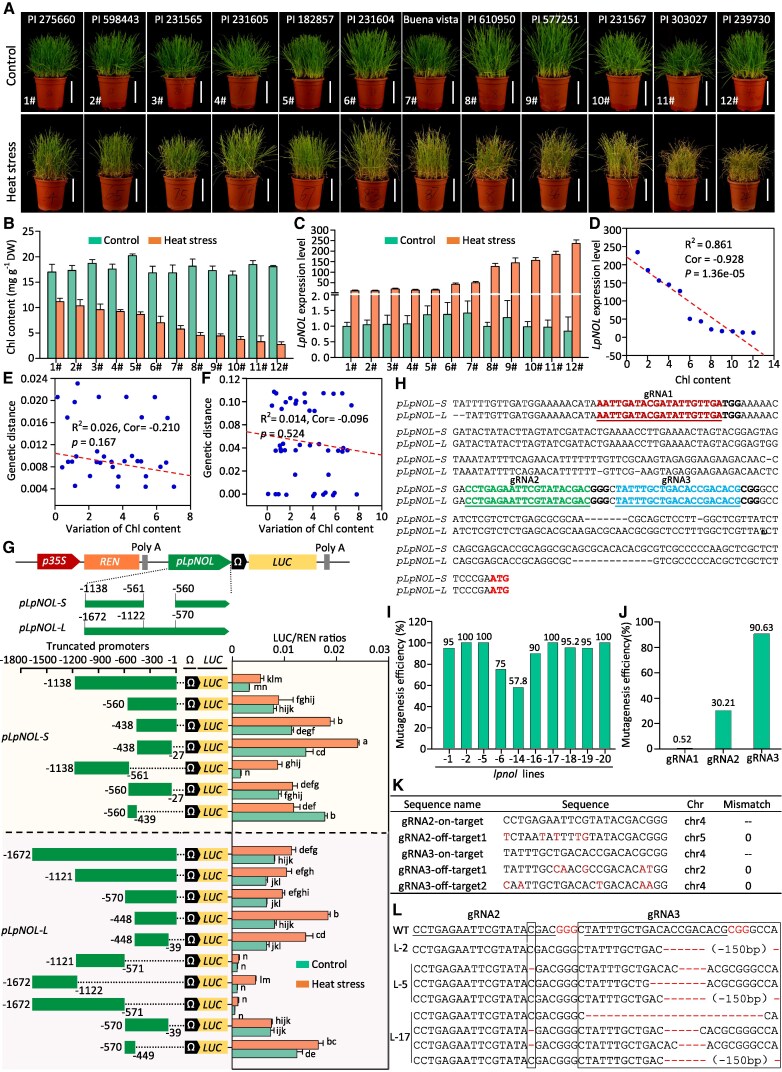
Targeted mutagenesis in *LpNOL* promoters in perennial ryegrass. **A** to **C)** Phenotypes, chlorophyll (Chl) content, and relative expression levels of *LpNOL* in 12 ryegrass varieties under optimum (control) or heat stress conditions. **D)** Correlation (Cor) analysis between the relative expression of *LpNOL* and Chl content of 12 ryegrass accessions under heat stress treatment. **E** to **F)** Correlation analysis between the variation of Chl content and the genetic distance of *pLpNOL*-S (E) or *pLpNOL*-*L* (F) between each pair of ryegrass accessions. The genetic distance between *pLpNOL*-S or *pLpNOL*-*L* of each pair of ryegrass varieties were calculated by MEGA 12 (Kumar et al. 2024). The variation of the Chl content was the absolute value of subtraction between 2 varieties. **G)** Truncated promoter analysis. The truncated *pLpNOL-S* or *pLpNOL-L* were used to drive the reporter gene *LUC* with *35S*::*REN* as the internal reference under optimal or heat stress conditions. Relative LUC/REN ratios represented promoters' transcriptional activities. **H)** Selection of gRNA targets in the consensus sequences of *pLpNOL-S* & *pLpNOL-L*. **I** to **J)** Mutagenesis efficiency of 10 CRISPR lines and 3 gRNAs. **K)** Predicted potential off-targets of gRNA2 and gRNA3. **L)** Mutations in *lpnol-2* (L-2), *−5* (L-5), and *−17* (L-17) lines. WT indicates wild type perennial ryegrass. Scale bars in (A) represent 10 cm. Data were subjected to statistical analysis by the Duncan test at a significance level of 0.05. Data in (B, C, and G) are means ± SE (*n* ≥ 3); different letters represent statistically significant differences at *P* ≤ 0.05.

We cloned the promoter of *LpNOL* (*pLpNOL*) from the 12 PR varieties and found that all of them have 2 types of *pLpNOLs* differing for the presence of a ∼552 bp fragment or not, therefore named these promoters as *pLpNOL-S* and *-L*, respectively (Supplementary Fig. S1 and Supplementary Text S1; see Supplementary Materials and Methods). However, the genetic distances among *pLpNOLs* were not correlated with heat-induced Chl loss of these PR varieties (Fig. 1, E and F; Supplementary Fig. S2), suggesting that the genetic diversity of *pLpNOLs* could not interpret the differential heat tolerance among the PR varieties.

Using *in silico* MEME motif and *cis*-element analyses, we found 2 conserved motifs on *pNOLs* of 4 different grass species (Supplementary Fig. S3). These motifs of *pLpNOLs* contain stress-related *cis*-elements, such as W-box (TTGAC, potential binding site by WRKYs) and CCGAC (potential binding site of DREB/CBF) (Supplementary Fig. S3). Multiple light-responsive *cis*-elements (CACGTG) were identified in the ∼552 bp fragment specific to *pLpNOL-L* suggesting that this fragment might regulate *LpNOL*'s expression through light-responsive signaling. Accordingly, we truncated *pLpNOL-S* into 6 fragments and *pLpNOL-L* into 9 fragments to test their transcriptional efficiencies under normal or high temperatures (Fig. 1G). Relative expression of the reporter gene (indicated by LUC/REN) driven under the truncated *pLpNOL-S*_-27∼-438_ and *pLpNOL-L*_-1∼-448_ had the highest transcriptional activities under high temperatures (Fig. 1G). Therefore, we designed 3 guide RNAs (gRNAs) targeting the consensus sequence of *pLpNOL-L* and *-S* close to the start codon (Fig. 1H), among which gRNA2 and gRNA3 were designed to target the conserved motif 1 (Supplementary Fig. S3). A multiplex gene editing vector was constructed using the polycistronic *tRNA-gRNA* (PTG) strategy (Supplementary Fig. S4) according to Yao et al. (2023).

Next, T_0_ generation of targeted *lpnol* mutants were generated using *Agrobacterium tumefaciens-*mediated genetic transformation following the protocol described in the Supplementary Materials and Methods. Twenty independent transgenic lines were obtained. Ten of them with obvious stay-green phenotypes were verified by PCR for the presence of the PTG cassette in their genomes (Supplementary Fig. S4). Their mutagenesis efficiencies were estimated to be 58% to 100% by sequencing the PCR amplicons of *LpNOL* (Fig. 1I). The mutagenesis efficiencies among the 3 targeted sites were 0.52%, 30.21%, and 90.63% by gRNA1, gRNA2, and gRNA3, respectively (Fig. 1J). Potential off-target sites of the 3 gRNAs were predicted based on the draft genome sequence of PR (Nagy et al. 2022). Sanger sequencing of the PCR amplicons of the 3 potential off-target sites didn’t reveal any mutation from 3 homozygous mutants (Fig. 1K; Supplementary Fig. S5), suggesting that the off-target event should be close to zero. Three homozygous mutant lines (*lpnol*-2, −5, and −17) with various point mutations and deletions (Fig. 1L) were used for phenotypic scoring.

As stated previously, the *NOLi* lines had significantly fewer tillers. Yet, the *lpnol* mutants had similar tiller numbers to wild type (WT) (Fig. 2, A and B). We reasoned that, in *NOLi*, the hairpin RNAi fragment contained nucleotides coding the conserved short-chain dehydrogenase/reductase domain that could abolish NOL's enzymatic activity (Yu et al. 2021). While, in the *lpnol* mutants, promoter deletion near the start codon did not abolish the gene but only decreased its expression levels. Nevertheless, both *lpnol* mutants and *NOLi* showed the typical stay-green phenotype: both had more numbers of green leaves in each tiller, slower dark-induced leaf senescence rate, and lower *LpNOL* expression levels (Fig. 2, C to I). *lpnol* mutants had shorter leaves and reduced biomass yield than WT under the optimal growth temperature (Fig. 2C; Supplementary Fig. S6). Under heat stress, both *lpnol* and *NOLi* showed higher heat tolerance with significantly more Chl contents, less membrane damage of leaf cells (lower electrolyte leakage rates), and less heat-induced biomass reductions than WT (Fig. 2, J to M; Supplementary Fig. S6).

**Figure 2.**
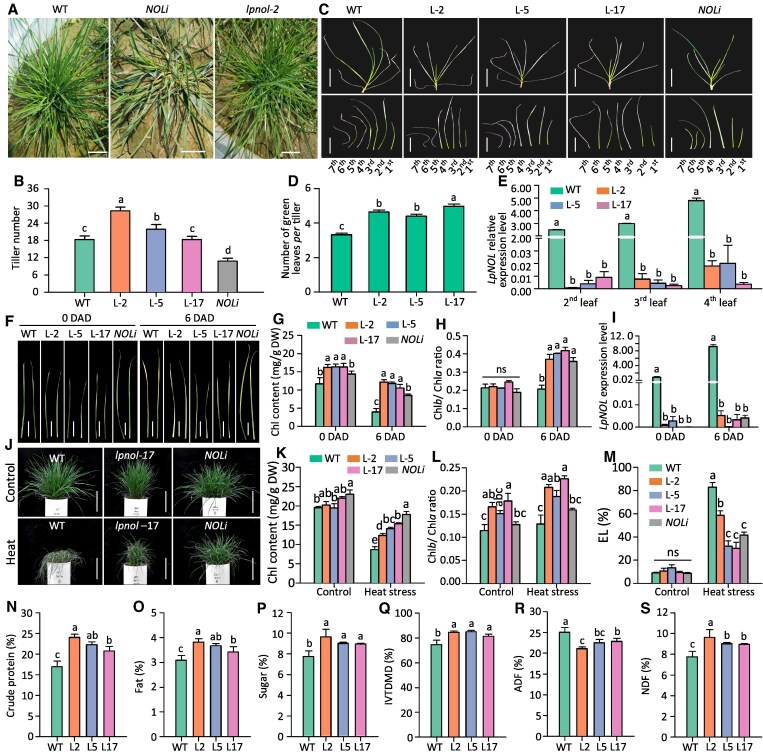
Mutagenesis in *LpNOL* promoters resulted in stay-green trait, increased forage quality, and improved heat tolerance. **A** to **B)** Constitutive RNAi *LpNOL* (*NOLi*) reduced tillering capacity, while *lpnol* with mutated *pLpNOLs* had uncompromised tillering. **C** to **E)** The *lpnol* CRISPR lines (L-2, L-3, and L-17) had more green leaf numbers than WT (wild type) with significantly lower expression levels of *LpNOL* in the 2nd-4th leaves from the top. **F** to **I)** Leaves of *lpnol* CRISPR lines demonstrated the stay-green trait like that of *NOLi*. After 6 d of dark treatment, leaves of both *lpnol* and *NOLi* lines remained greener than those of WT (F) with significantly higher Chl contents (G), higher Chl*b*/Chl*a* ratios (H), and lower *LpNOL* expression levels (I). **J** to **M)**  *lpnol* lines were more heat tolerant with higher Chl contents (J to L) and less electrolyte leakage (EL) rates than WT (M). **N** to **S)**  *lpnol* lines had higher crude protein, fat, and sugar contents, higher in vitro dry matter digestibility (IVTDMD), less acid detergent fiber, and higher neutral detergent fiber contents than WT. Images in (C and F) were digitally extracted for comparison. Scale bars in (A) represent 5 cm; scale bars in (C and F) represent 2.5 cm; scale bars in (J) represent 10 cm. Data were subjected to statistical analysis by the Duncan test at a significance level of 0.05. All quantitative data in this figure are means ± SE (*n* = 3); different letters represent statistically significant differences at *P* ≤ 0.05.

Furthermore, the stay-green *lpnol* mutants also had significantly improved forage quality traits, including up to 41.3% higher protein content, 23.3% higher fat content, 24.3% higher sugar content, and 13.8% higher in vitro dry matter digestibility under the greenhouse-growth condition (Fig. 2, N to S; Supplementary Fig. S7). These improved forage quality traits might be due to the higher photosynthesis capacity of the functional stay-green leaves and enhanced stability of Chl-binding proteins (Yu et al. 2021). Further field-based tests are necessary to evaluate the application potential of the stay-green phenotype, improved heat-tolerance, and higher forage qualities in PR breeding programs.

However, the *lpnol* lines did not sprout any reproductive tiller or flower while the WT flowered normally in the late spring for 2 yr (Supplementary Fig. S8). Similarly, over-expressing the Chl*b* synthesis enzymatic gene, *chlorophyllide a oxygenase*, resulted in Chl*b* accumulation and delayed flowering (>50% d to anthesis) in tobacco (*Nicotiana tabacum*) under high light conditions (Biswal et al. 2012). Chl*b* might influence flowering time regulators like CONSTANS (CO) through photoreceptor (phytochrome and cryptochrome)-mediated light signaling. It's also possible that blocking Chl*b* catabolism affected the metabolism of tetrapyrroles (precursors of both Chl and phytochromobilin). Land plants' photoreceptors use phytochromobilin as chromophore to sense light signals. It's possible that Chl*b* metabolism indirectly affect chromophores and thereby the light signaling for anthesis. If the mutant could flower in the higher latitude regions with longer daytime for seed production, ryegrass with the abolished flowering trait grown in transition zones could be valued for easier grassland/turf maintenance. Further study on *NOLs* is necessary to clarify its impact on flowering in the future.

In summary, this study demonstrated that rationally designed promoter editing could retain the desirable traits (i.e. stay-green and improved heat tolerance) while avoiding the side effects of complete gene knock-out/down (i.e. reduced tillering). Similarly, removal of transcription repression motifs in the promoters of *NF-YC4* in rice (*Oryza sativa*) and soybean (*Glycine max*) significantly increased gene expression levels and seed protein contents (Wang *et al*. 2025). Combined with the increasing knowledge of senescence-associated genes (Guo et al. 2025), targeted promoter mutations provide a savvy approach for designed molecular breeding in ryegrass and other plant species.

## Supplementary Material

kiaf402_Supplementary_Data

## Data Availability

Data are available in the online version of this article.

## Supplementary data

The following materials are available in the online version of this article.

The following materials are available in the online version of this article.

**Supplementary Figure S1.** Sequence alignment of short (*pLpNOL-S*) and long (*pLpNOL-L*) *LpNOL* promoters of perennial ryegrass cv. “Buena vista”.

**Supplementary Figure S2.** Phylogenetic analysis of short (*pLpNOL-S*) and long *LpNOL* promoters (*pLpNOL-L*) *LpNOL* promoters in perennial ryegrass accessions.

**Supplementary Figure S3.**  *In silico* analysis of the consensus motifs among the *NOL* promoters of perennial ryegrass, barley (*Hordeum vulgare*, *HvNOL*), rice (*Oryza sativa*, *OsNOL*), and switchgrass (*Panicum virgatum*, *PvNOL*).

**Supplementary Figure S4.** Diagram of the CRISPR vector used for *p*-*LpNOL* knockout and PCR verification of the PTG cassette in the T0 generation of *lpnol* CRISPR lines.

**Supplementary Figure S5.** Sanger sequencing of PCR products harboring potential off-target sites.

**Supplementary Figure S6.** Biomass of WT, *lpnol* CRISPR-edited lines, and *LpNOLi* lines under control and heat stress conditions.

**Supplementary Figure S7.** Ash and lignin content in wild type (WT) and *lpnol* CRISPR-edited lines (L-2, L-5, and L-17).

**Supplementary Figure S8.** The *lpnol* mutants do not undergo anthesis.

**Supplementary Table S1.** Primers used in RT-qPCR.

**Supplementary Table S2.** Primers used in PCR for the evaluation of potential off-targets.

**Supplementary Text S1.** Sequences of *pLpNOL-L* and *pLpNOL-S* of tested varieties of perennial ryegrass.

## Dive Curated Terms

The following phenotypic, genotypic, and functional terms are of significance to the work described in this paper:

NF-YC4 Gramene: AT5G63470
NF-YC4 Araport: AT5G63470

MEGA CHEBI: CHEBI:6617
