# Supplementary material for: Promoter editing of LpNOL generates stay-green perennial ryegrass with improved forage quality and heat tolerance
Source: Plant Physiol. 2025 Sep 30;199(2):kiaf402. doi: 10.1093/plphys/kiaf402 (PMC12483074; doi:10.1093/plphys/kiaf402)
Supplement: kiaf402_Supplementary_Data [file kiaf402_supplementary_data.pdf]

# Supplementary Figures

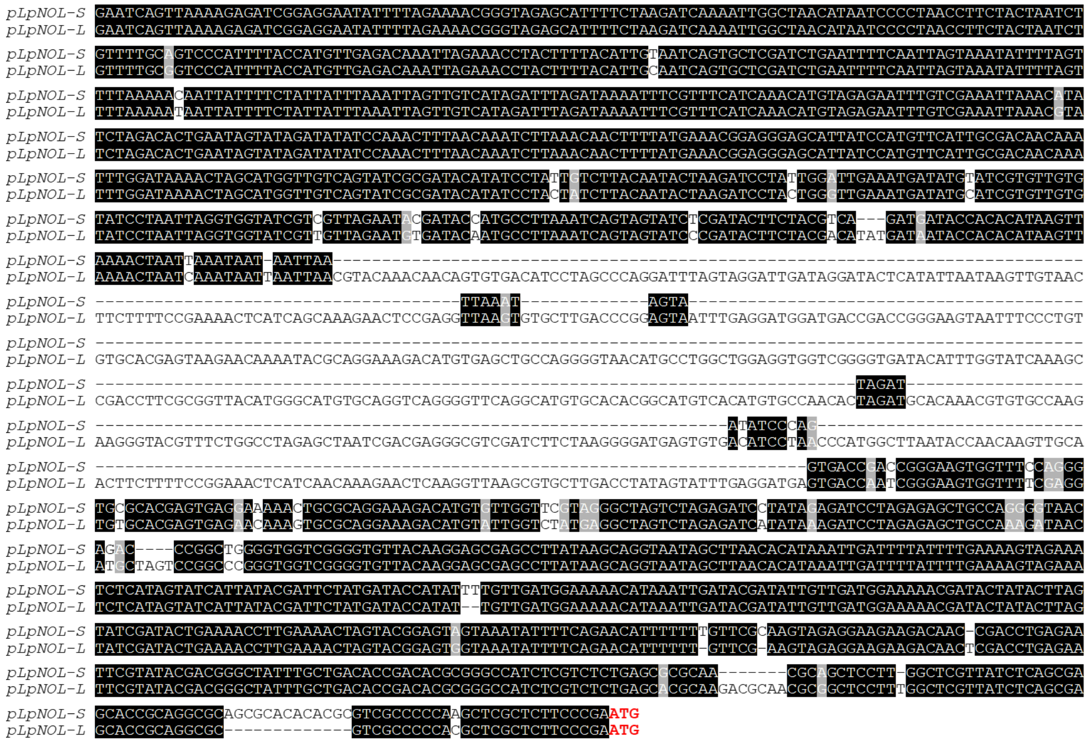

**Supplementary Figure S1**. Sequence alignment of short (*pLpNOL-S*) and long (*pLpNOL-L*) *LpNOL* promoters of perennial ryegrass cv. 'Buena vista'. Nucleic acids in black are identical, and those in grey are homologous base changes.

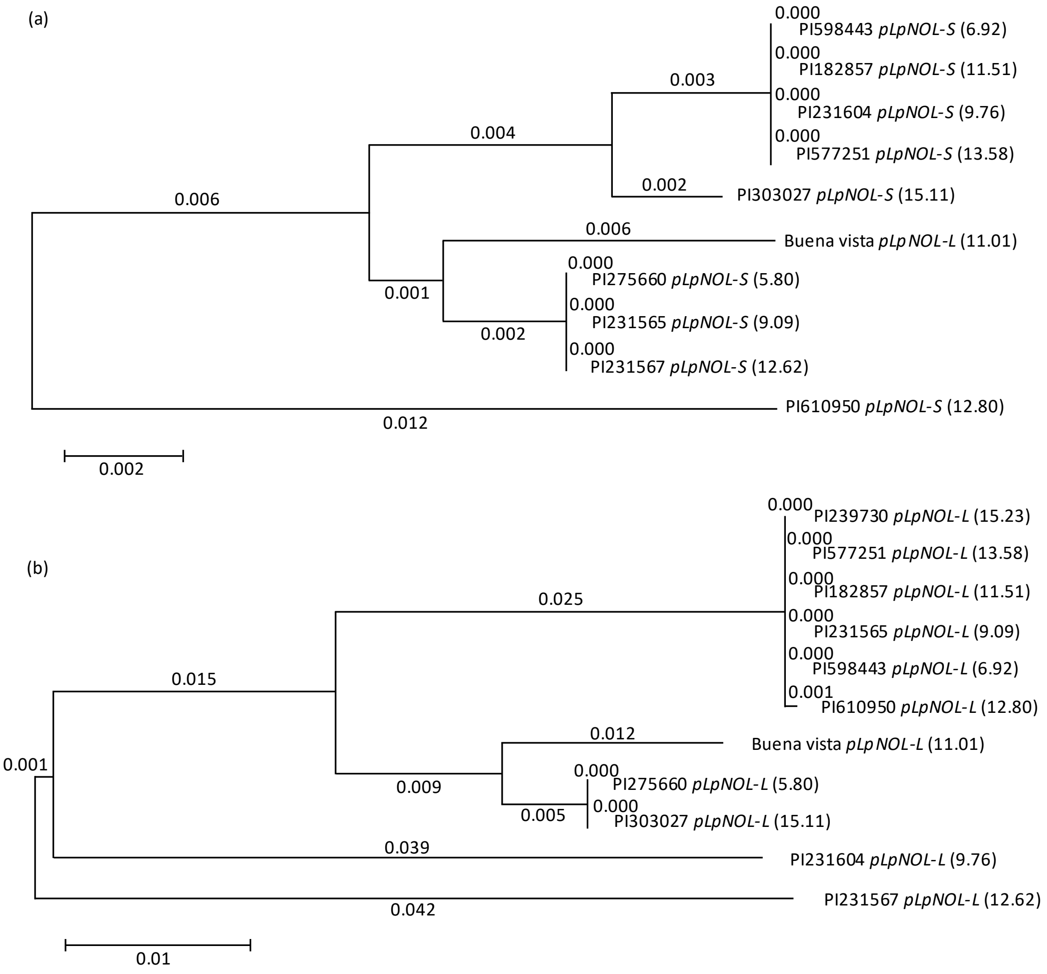


**Supplementary Figure S2**. Phylogenetic analysis of short (*pLpNOL-S*) and long (*pLpNOL-L*) *LpNOL* promoters in perennial ryegrass accessions. (A) Phylogenetic analysis of *pLpNOL-S*; (B) Phylogenetic analysis of *pLpNOL-L*. The genetic distances among these *pLpNOLs* are shown above or below the branches. The numbers in parentheses following the *pLpNOL-S* or -*L* are the differences between the chlorophyll content [mean value, $mg\cdot g^{-1}$ (DW, dry weight)] of each ryegrass germplasm under control and that under heat stress conditions. The evolutionary history was inferred by using the Maximum Likelihood method. The tree is drawn to scale, with branch lengths measured in the number of substitutions per site (next to the branches). Evolutionary analyses were conducted in MEGA 12.

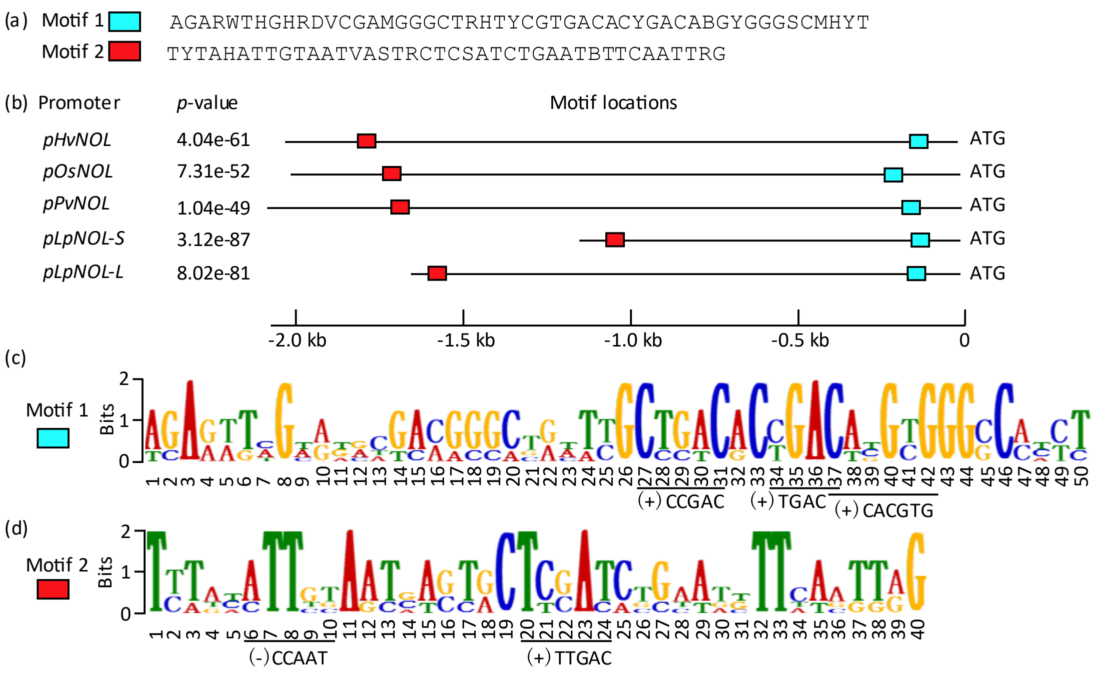


**Supplementary Figure S3**. *In silico* analysis of the consensus motifs among the *NOL* promoters of perennial ryegrass, barley (*Hordeum vulgare*, *HvNOL*), rice (*Oryza sativa*, *OsNOL*), and switchgrass (*Panicum virgatum*, *PvNOL*). (+) represents the forward sequence and (-) represents the reverse complementary sequence. Among the motif 1, there are CCAAT *cis*-element (a ubiquitous cis-element of eukaryotic promoters that is present in ~ 30% of eukaryotic genes' promoters primarily activated by conserved CCAAT-binding complexes) and a W-box (TTGAC) *cis*-element potentially recognized by WRKY family transcription factors. In the motif 2, there are the CCGAC *cis*-element (the binding site of DREB/CBF) and G-box (CACGTG) *cis*-element (a ubiquitous regulatory DNA element in plants bound by the G-box factors of bZIP proteins).

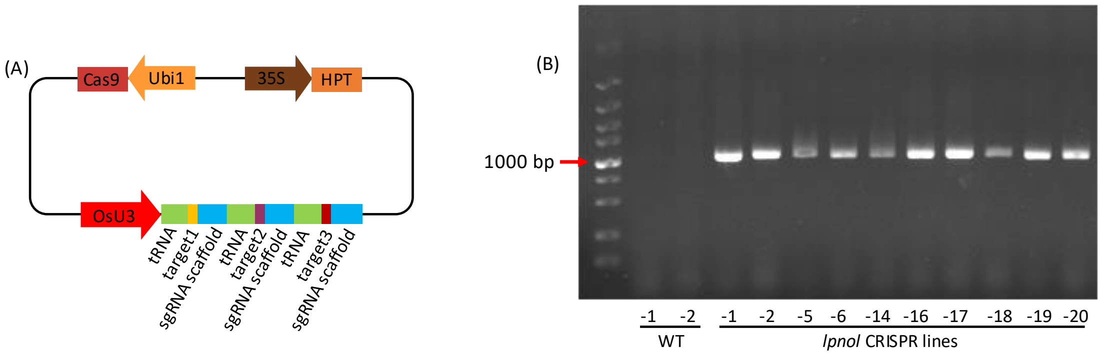


**Supplementary Figure S4**. Diagram of the CRISPR vector used for *p-LpNOL* knockout and PCR verification of the PTG cassette in the T0 generation of *lpnol* CRISPR lines. (A) CRISPR vector diagram; (B) Electrophoresis of the PCR products of WT (wild type) and *lpnol*-CRISPR lines. The red arrow in (B) indicates predicated size of the PCR product.

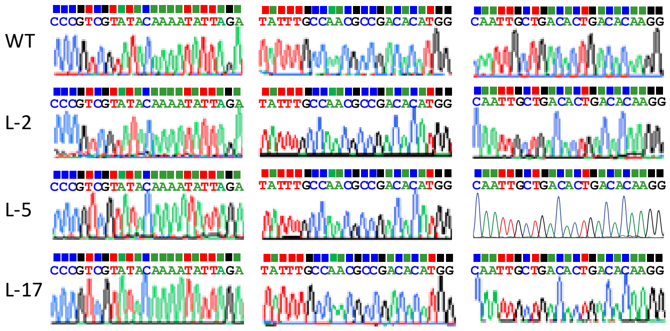


**Supplementary Figure S5**. Sanger sequencing of PCR products harboring potential off-target sites. WT indicates wild type and L-2, L-5, and L-17 are *lpnol*-CRISPR lines.

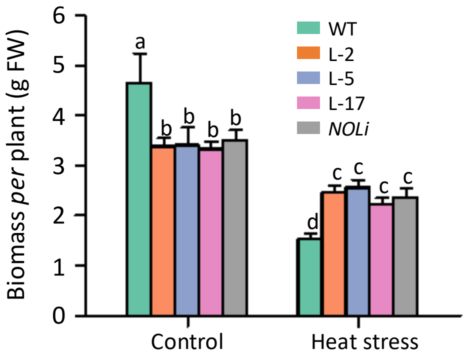


**Supplementary Figure S6**. Biomass of WT (wild type), *lpnol* CRISPR-edited lines (L-2, L-5, and L-17), and *LpNOL* RNAi (*NOLi*) lines under control and heat stress conditions. The WT, *lpnol* mutants and *LpNOLi* transgenic ryegrass were placed in a constant-temperature water baths, where both aerial and root temperatures were set at 25°C or 35°C for 4 days, and then recovered for 5 days at 25°C for 5 days. Then, their above-ground biomasses were harvested for fresh weights (FW) measurement. Data were subjected to statistical analysis by the Duncan test at a significance level of 0.05. Data are means ± SE (n=3); different letters represent statistically significant differences at p≤0.05.

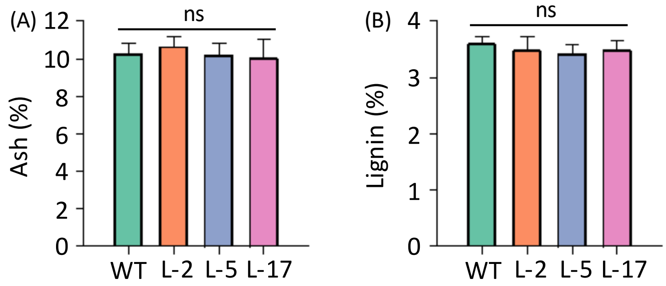


**Supplementary Figure S7**. Ash and lignin content in wild type (WT) and *lpnol* CRISPR-edited lines (L-2, L-5, and L-17). Error bars represent standard errors. No significant differences were detected in ash and lignin content among WT and *lpnol* CRISPR-edited lines. Data were subjected to statistical analysis by the Duncan test at a significance level of 0.05. Data are means ± SE (n=3); 'ns' indicates no statistically significant differences at $p \leq 0.05$.

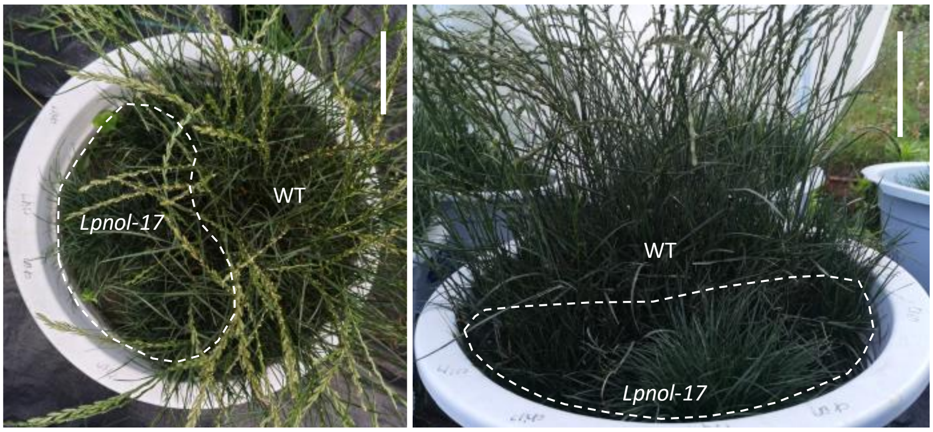


**Supplementary Figure S8**. The *lpnol* mutants do not undergo anthesis. The wild type (WT), three mutant lines (*lpnol-2*, *lpnol-5*, *lpnol-17*), and *LpNOL*-RNAi (*NOLi*) ryegrass lines were grown in big pots (diameter of 70 cm) to allow sufficient vegetative growth in Nanjing, China (118°796'E, 32°048'N). These plants were vernalized over-winter. For two years, the *lpnol* mutant and *NOLi* ryegrass lines did not have reproductive tiller, while the WT flowered normally. Scale bars represent 10 cm.

**Supplementary Table S1.** Primers used in RT-qPCR

| Primers | Sequence（5΄-3΄） |
|---|---|
| *LpNOL*-qRTF | GCTGGCAAAGAAGTTTCTCA |
| *LpNOL*-qRTR | ATGCTGCTCTCCAAATTCCT |
| *LpeIF4A*-qRTF | AACTCAACTTGAAGTGTTGGAGTG |
| *LpeIF4A*-qRTR | AGATCTGGTCCTGGAAAGAATATG |

**Supplementary Table S2.** Primers used in PCR for the evaluation of potential off-targets

| Primers | Sequence（5΄-3΄） |
|---|---|
| gRNA2-off-target1-FP | TCCTTGCGGGAAAACCTC |
| gRNA2-off-target1-RP | GATGGCGAGTGCTCTGTG |
| gRNA3-off-target1-FP | TCTGCTACGCATGCACTG |
| gRNA3-off-target1-RP | CGCCCTCTCCGAACAAGA |
| gRNA3-off-target2-FP | AGACCACACTACTACTGGATACTC |
| gRNA3-off-target2-RP | TGTGCAGGTACATGCGTAAGA |
| *LpNOL*-gRNAs-FP | GCAGGAAAGACATGTGTTG |
| *LpNOL*-gRNAs-RP | TGATGAGGACATTGTACGG |

**Supplementary Text S1.** Sequences of *pLpNOL-L* and *pLpNOL-S* of tested varieties of perennial ryegrass.

```
> Buena Vista-S
GAATCAGTTAAAAGAGATCGGAGGAATATTTTAGAAAACGGGTAGAGCATTTTCTAAGATCAAAATTGGCTAACATAATCCCCTAA
CCTTCTACTAATCTGTTTTGCAGTCCCATTTTACCATGTTGAGACAAATTAGAAACCTACTTTTACATTGTAATCAGTGCTCGATC
TGAATTTTCAATTAGTAAATATTTTAGTTTTAAAAACAATTATTTTCTATTATTTAAATTAGTTGTCATAGATTTAGATAAAATTT
CGTTTCATCAAACATGTAGAGAATTTGTCGAAATTAAACATATCTAGACACTGAATAGTATAGATATATCCAAACTTTAACAAATC
TTAAACAACTTTTATGAAACGGAGGGAGCATTATCCATGTTCATTGCGACAACAAATTTGGATAAAACTAGCATGGTTGTCAGTAT
CGCGATACATATCCTATTGTCTTACAATACTAAGATCCTATTGGATTGAAATGATATGTATCGTGTTGTGTATCCTAATTAGGTGG
TATCGTCGTTAGAATACGATACCATGCCTTAAATCAGTAGTATCTCGATACTTCTACGTCAGATGATACCACACATAAGTTAAAAC
TAATTAAATAATAATTAATTAAATAGTATAGATATATCCCAGGTGACCGACCGGGAAGTGGTTTCCAGGGTGCGCACGAGTGAGGA
AAAACTGCGCAGGAAAGACATGTGTTGGTTCGTAGGGCTAGTCTAGAGATCCTATAGAGATCCTAGAGAGCTGCCAGGGGTAACAG
ACCCGGCTGGGGTGGTCGGGGTGTTACAAGGAGCGAGCCTTATAAGCAGGTAATAGCTTAACACATAAATTGATTTTATTTTGAAA
AGTAGAAATCTCATAGTATCATTATACGATTCTATGATACCATATTTTGTTGATGGAAAAACATAAATTGATACGATATTGTTGAT
GGAAAAACGATACTATACTTAGTATCGATACTGAAAACCTTGAAAACTAGTACGGAGTAGTAAATATTTTCAGAACATTTTTTTGT
TCGCAAGTAGAGGAAGAAGACAACCGACCTGAGAATTCGTATACGACGGGCTATTTGCTGACACCGACACGCGGGCCATCTCGTCT
CTGAGCGCGCAACGCAGCTCCTTGGCTCGTTATCTCAGCGAGCACCGCAGGCGCAGCGCACACACGCGTCGCCCCCAAGCTCGCTC
TTCCCGA

>Buena Vista-L
TTGGCTAACATAATCCCCTAACCTTCTACTAATCTGTTTTGCGGTCCCATTTTACCATGTTGAGACAAATTAGAAACCTACTTTTA
CATTGCAATCAGTGCTCGATCTGAATTTTCAATTAGTAAATATTTTAGTTTTAAAAATAATTATTTTCTATTATTTAAATTAGTTG
TCATAGATTTAGATAAAATTTCGTTTCATCAAACATGTAGAGAATTTGTCGAAATTAAACGTATCTAGACACTGAATAGTATAGAT
```

ATATCCAAACTTTAACAAATCTTAAACAACTTTTATGAAACGGAGGGAGCATTATCCATGTTCATTGCGACAACAAATTTGGATAA
AACTAGCATGGTTGTCAGTATCGCGATACATATCCTACTATCTTACAATACTAAGATCCTACTGGGTTGAAATGATATGCATCGTG
TTGTGTATCCTAATTAGGTGGTATCGTTGTTAGAATGTGATACAATGCCTTAAATCAGTAGTATCCCGATACTTCTACGACATATG
ATAATACCACACATAAGTTAAAACTAATCAAATAATTAATTAACGTACAAACAACAGTGTGACATCCTAGCCCAGGATTTAGTAGG
ATTGATAGGATACTCATATTAATAAGTTGTAACTTCTTTTCCGAAAACTCATCAGCAAAGAACTCCGAGGTTAAGTGTGCTTGACC
CGGAGTAATTTGAGGATGGATGACCGACCGGGAAGTAATTTCCCTGTGTGCACGAGTAAGAACAAAATACGCAGGAAAGACATGTG
AGCTGCCAGGGGTAACATGCCTGGCTGGAGGTGGTCGGGGTGATACATTTGGTATCAAAGCCGACCTTCGCGGTTACATGGGCATG
TGCAGGTCAGGGGTTCAGGCATGTGCACACGGCATGTCACATGTGCCAACACTAGATGCACAAACGTGTGCCAAGAAGGGTACGTT
TCTGGCCTAGAGCTAATCGACGAGGGCGTCGATCTTCTAAGGGGATGAGTGTGACATCCTAACCCATGGCTTAATACCAACAAGTT
GCAACTTCTTTTCCGGAAACTCATCAACAAAGAACTCAAGGTTAAGCGTGCTTGACCTATAGTATTTGAGGATGAGTGACCAATCG
GGAAGTGGTTTTCGAGGTGTGCACGAGTGAGAACAAAGTGCGCAGGAAAGACATGTATTGGTCTATGAGGCTAGTCTAGAGATCAT
ATAAAGATCCTAGAGAGCTGCCAAAGATAACATGCTAGTCCGGCCCGGGTGGTCGGGGTGTTACAAGGAGCGAGCCTTATAAGCAG
GTAATAGCTTAACACATAAATTGATTTTATTTTGAAAAGTAGAAATCTCATAGTATCATTATACGATTCTATGATACCATATTGTT
GATGGAAAAACATAAATTGATACGATATTGTTGATGGAAAAACGATACTATACTTAGTATCGATACTGAAAACCTTGAAAACTAGT
ACGGAGTGGTAAATATTTTCAGAACATTTTTTGTTCGAAGTAGAGGAAGAAGACAACTCGACCTGAGAATTCGTATACGACGGGCT
ATTTGCTGACACCGACACGCGGGCCATCTCGTCTCTGAGCACGCAAGACGCAACGCGGCTCCTTTGGCTCGTTATCTCAGCGAGCA
CCGCAGGCGCGTCGCCCCCACGCTCGCTCTTCCCGA

>PI275660-S
TTGGCTAACATAATCCCCTAACCTTCTACTAATCTGTTTTGCAGTCCCATTTTACCATGTTGAGACAAATTAGAAACCTACTTTTA
CATTGTAATCAGTGCTCGATCTGAATTTTCAATTAGTAAATATTTTAGTTTTAAAAACAATTATTTTCTATTATTTAAATTAGTTG
TCATAGATTTAGATAAAATTTCGTTTCATCAAACATGTAGAGAATTTGTCGAAATTAAACATATCTAGACACTGAATAGTATAGAT
ATATCCAAACTTTAACAAATCTTAAACAACTTTTATGAAACGGAGGGAGCATTATCCATGTTCATTGCGACAACAAATTTGGATAA
AACTAGCATGGTTGTCAGTATCGCGATACATATCCTATTGTCTTACAATACTAAGATCCTATTGGGTTGAAATGATATGTATCGTG
TTGTGTATCCTAATTAGGTGGTATCGTCGTTAGAATACGATACCATGCCTTAAATCAGTAGTATCTCGATACTTCTACGTCAGATG
ATACCACACATAAGTTAAAACTAATTAAATAATAATTAATTAAATAGTATAGATATATCCCAGGTGACCGACCGGGAAGTGGTTTC
CAGGGTGCGCACGAGTGAGGAAAAACTGCGTAGGAAAGACATGTGTTGGTTCGTAGGGCTAGTCTAGAGATCCTATAGAGATCCTA
GAGAGCTGCCAGGGGTAACAGACCCGGCTGGGGTGGTCGGGGTGTTACAAGGAGCGAGCCTTATAAGCAGGTAATAGCTTAACACA
TAAATTGATTTTATTTTGAAAAGTAGAAATCTCATAGTATCATTATACGATTCTATGATACCATATTGTTGATGGAAAAACATAAA
TTGATACGATATTGTTGATGGAAAAACGATACTATACTTAGTATCGATACTGAAAACCTTGAAAACTAGTACGGAGTGGTAAATAT
TTTCAGAACATTTTTTGTTCGAAGTAGAGGAAGAAGACAACTGGACCTGAGAATTCGTATACGACGGGCTATTTGCTGACACCGAC
ACGCGGGCCATCTCGTCTCTGAGCACGCAAGACGCAGCGCGGCTCCTTGGCTCGTTATCTCAGCGAGCACCGCAGCTCACACACGC
GTCGCCCCCAAGCTCGCTCTTCCCGA**ATG**

> PI275660-L
TTGGCTAACATAATCCCCTAACCTTCTACTAATCTGTTTTGCAGTCCCATTTTACCATGTTGAGACAAATTAGAAACCTACTTTTA
CATTGTAATCAGTGCTCGATCTGAATTTTCAATTAGTAAATATTTTAGTTTTAAAAATAATTATTTTCTATTATTTAAATTAGTTG
TCATAGATTTAGATAAAATTTCGTTTCATCAAACATGTAGAGAATTTGTCGAAATTAAACGTATCTAGACACTGAATAGTATAGAT
ATATCCAAACTTTAACAAATCTTAAACAACTTTTATGAAACGGAGGGAGCATTATCCATGTTCATTGCGACAACAAATTTGGATAA
AACTAGCATGGTTGTCAGTATCGCGATACATATCCTACTATCTTACAATACTAAGATCCTACTGGGTTGAAATGATATGCATCGTG
TTGTGTATCCTAATTAGGTGGTATCGTTGTTAGAATGTGATACAATGCCTTAAATCAGTAGTATCCCGATACTTCTACGACATATG
ATAATACCACACATAAGTTAAAACTAATCAAATAATTAATTAACGTACAAACAACAGTGTGACATCCTAGCCCAGGATTTAGTAGG
ATTGATAGGATACTCATATTAATAAGTTGTAACTTCTTTTCCGAAAACTCATCAGCAAAGAACTCCGAGGTTAAGTGTGCTTGACC
CGGAGTAATTTGAGGATGGATGACCGACCGGGAAGTAGTTTCCCTGTGTGCACGAGTGAGAACAAAATACGCAGGAAAGACATGTG
AGCTGCCAGGGATAACATGCCTGGCTGGAGGTGGTCGGGGTGATACATTTGGTATCAAAGCCGACCTTCGCGGTTACATGGACATG
TGCAGGTCAGGGGTTCAGGCATGTGCACACGGCATGTCACATGTGCCAACACTAGATGCACAAACGTGTGCCAAGAAGGGTACGTT
TCTGGCCTAGAGCTAATCGACGAGGGCGTCGATCTTCTAAGGGGATGAGTGTGACATCCTAGCCCATGGCTTAATACCAACAAGTT
GCAACTTCTTTTCCGGAAACTCATCAACAAAGAACTCGAGGTTAAGCATGCTTGACCTATAGTATTTGAGGATGAGTGACCGATCG
GGAAGTGGTTTTCGAGGTGTGCACGAGTGAGAACAAAATGCGCAGGAAAGACATGTGTTGGTCTATGAGGCTAGTCTAGAGATCAT
ATAAAGATCCTAGAGAGCTGCCACAGATAACGGGCCGGTCCGACCAGGGTGATCGGGGTGTTACAAGGAGCGAGCCTTATAAGCAG
GTAATAGCTTAACACATAAATTGATTTTATTTTGAAAAGTAGAAAATCTCATAGTATCATTATACGATTCTATGATACCATATTTT
GTTGATGGAAAAAACATAAATTGATACGATATTGTTGATGGAAAAACGATACTATACTTAGTATCGATACGGAAAACCTTGAAATT
TAGTACGGAGTAGTAAATATTTTCAGAACATTTTTTGTTCGCAAGTAGAGGAAGAAGACAACTCGACCTGAGAATTCGTATACGAC
GGGCTATTTGCTGACACCGACACGCGGGCCATCTCGTCTCTGAGCACGCAACGCAGCTCCTTGGCTCGTTATCTCAGCGACAGCGA
GCACCGCAGCGCGCACACGCGTCGCCCCCAAGCTCGCTCTTCCCGA**ATG**

>PI598443-S
TTGGCTAACATAATCCCCTAACCTTCTACTAATCTGTTTTGCAGTCCCATTTTACCATGTTGAGACAAATTAGAAACCTACTTTTA
CATTGTAATCAGTGCTCGATCTGAATTTTCAATTAGTAAATATTTTAGTTTTAAAAACAATTATTTTCTATTATTTAAATTAGTTG
TCATAGATTTAGATAAAATTTCGTTTCATCAAACATGTAGAGAATTTGTCGAAATTAAACATATCTAGACACTGAATAGTATAGAT
ATATCCAAACTTTAACAAATCTTAAACAACTTTTATGAAACGGAGGGAGCATTATCCATGTTCATTGCGACAACAAATTTGGATAA
AACTAGCATGGTTGTCAGTATCGCGATACATATCCTATTGTCTTACAATACTAAGATCCTATTGGATTGAAATGATATGTATCGTG
TTGTGTATCCTAATTAGGTGGTATCGTCGTTAGAATACGATACCATGCCTTAAATCAGTAGTATCTCGATACTTCTACGTCAGATG
ATACCACACATAAGTTAAAACTAATTAAATAATAATTAATTAAATAGTATAGATATATCCCAGGTGACCGACCGGGAAGTGGTTTC
CAGGGTGCGCACGAGTGAGGAAAAACTGCGCAGGAAAGACATGTGTTGGTTCGTAGGGCTAGTCTAAAGATCCTATAGAGATCCTA
GAGAGCTGCCAGGGGTAACAGACCCGGCTGGGGTGGTCGGGGTGTTACAAGGAGCGAGCCTTATAAGCAGGTAATAGCTTAACACA
TAAATTGATTTTATTTTGAAAAGTACAAATCTCATAGTATCATTATACGATTCTATGATACCATATTGTTGATGGAAAAACATAAA
TTGATACGATATTGTTGATGGAAAAACGATACTATACTTAGTATCGATACTGAAAACCTTGAAAACTAGTACGGAGTGGTAAATAT
TTTCAGAACATTTTTTGTTCGAAGTAGAGGAAGAAGACAACTCGACCTGAGAATTCGTATACGACGGGCTATTTGCTGACACCGAC
ACGCGGGCCATCTCGTCTCTGAGCACGCAAGACGCAACGCGGCTCCTTTGGCTCGTTATCTCAGCGAGCACCGCAGGCGCGTCGCC
CCCACGCTCGCTCTTCCCGA**ATG**

> PI598443-L
TTGGCTAACATAATCCCCTAACCTTCTACTAATCTGTTTTGCAGTCCCATTTTACCATGTTGAGACAAATTAGAAACCTACTTTTA
CATTGTAATCAGTGCTCGATCTGAATTTTCAATTAGTAAATATTTTAGTTTTAAAAACAATTATTTTCTATTATTTAAATTAGTTG
TCATAGATTTAGATAAAATTTCGTTTCATCAAACATGTAGAGAATTTGTCGAAATTAAACGTATCTAGACACTGAATAGTATAGAT
ATATTCAAACTTTAACAAATCTTAAACAACTTTTATGAAACGGAGGGAGCATTATCCATGTTCATTGCGACAACAAATTTGGATAA
AACTAGCATGGTTGTCCGCGATACATATCCTACTATCTTACAATACTAAGATCCTACTGGGTTGAAATGATATGTATCGTGTTGTG
TATCCTAATTAGGTGGTATCGTTGTTAGAATGTGATAACATGCCTTAAATCAGTAGTATCCCGATACTTCTACGACAGATGATACC
ACACATAAGTTAAAACTAATCAAATAATTAATTAACGTACAAACAAACAACAGTGTGACATCCTAGCCCAGGACTTAGTAGGATTG
ATAGGATACTCATATCAATAAGTTGTAACTTCTTTTCCGGAAATTCATCAGCAAACAACTCCGAGGTTAAGTGTGCTTGACCCGGA
GTAATTTGAGGATGGATGGCCGATCAGGAAGTAGTTTTCCTGTGTGCACGAGTGAGGACAAAATGCGCAGGAAAGACATGTGAGCT
GCGAGGGTAACATGCCCGGCCGGGGTGGTCGGGGTGATATATTTGGTATCAAAGCCGACCTTCGTGGTTACATGGGCATGTGCAGG
TCAGGGGTTCGGGTATGTGCACACGGCATGTCACATGTGCCAACACTGGATGCATAAACGTGTGCCAAGAAGGGTACGTTTCTGGC
CTAGAGCTAATCGACGAGTGTGTCGATCTTCTAAGGGGATGAGTGTGACATCCTAGCCCATGGCTTAATGCCAACAAGTTGTAACT
TCTTTTCCGGAAACTCATCAACAAAGAACTCCAAGGTTAAGCATGCTTGACCTTTTGTAATTTGAGGATGGGTAACCGATGGGGAA
GTGGTTTCCGAGGTGTGCACGAGTGAGAACAAAGTGCGCAGGAAAGACATGTGTTGGTCTATAAGGCTAGTCTAGAGATCATATAA
AGATCCTAGAGAGCTGCCGGCCGGTCCGACCGGGGTGATCGGGGTGTTACAAGGAGCGAGCCTTATAAGCAGGTAATAGCTTAACA
CATAAATTGATTTTATTTTGAAAAAAGTAGAAATCTCATAGTATCATTATACGATTCTATGATACCATATTGTTGATGGAAAAACA
TAAATTGATACGATATTGTTGATGGAAAAACGATACTATACTTAGTATCGATACGGAAAACCTTAAAAACTAGTACGGAGTAGTAA
ATATTTTCAGAACATTTTTTTGTTCGCAAGTAGAGGAAGAAGACAACTCGACCTGAGAATTCGTATACGACGGGCTATTTGCTGAC
ACCGACACGTGGGCCATCTCGTCTCTGAGCACGCAAGACGCAACGCGGCTCCTTGGCTCGTTATCTCAGCGAGCACCGCAGGCGCA
GCACACACACGCGTCGCCCCCAAGCTCGCTCTTCCCGA**ATG**

>PI231565-S
TTGGCTAACATAATCCCCTAACCTTCTACTAATCTGTTTTGCAGTCCCATTTTACCATGTTGAGACAAATTAGAAACCTACTTTTA
CATTGTAATCAGTGCTCGATCTGAATTTTCAATTAGTAAATATTTTAGTTTTAAAAACAATTATTTTCTATTATTTAAATTAGTTG
TCATAGATTTAGATAAAATTTCGTTTCATCAAACATGTAGAGAATTTGTCGAAATTAAACATATCTAGACACTGAATAGTATAGAT
ATATCCAAACTTTAACAAATCTTAAACAACTTTTATGAAACGGAGGGAGCATTATCCATGTTCATTGCGACAACAAATTTGGATAA
AACTAGCATGGTTGTCAGTATCGCGATACATATCCTATTGTCTTACAATACTAAGATCCTATTGGGTTGAAATGATATGTATCGTG
TTGTGTATCCTAATTAGGTGGTATCGTCGTTAGAATACGATACCATGCCTTAAATCAGTAGTATCTCGATACTTCTACGTCAGATG
ATACCACACATAAGTTAAAACTAATTAAATAATAATTAATTAAATAGTATAGATATATCCCAGGTGACCGACCGGGAAGTGGTTTC
CAGGGTGCGCACGAGTGAGGAAAAACTGCGTAGGAAAGACATGTGTTGGTTCGTAGGGCTAGTCTAGAGATCCTATAGAGATCCTA
GAGAGCTGCCAGGGGTAACAGACCCGGCTGGGGTGGTCGGGGTGTTACAAGGAGCGAGCCTTATAAGCAGGTAATAGCTTAACACA
TAAATTGATTTTATTTTGAAAAGTAGAAATCTCATAGTATCATTATACGATTCTATGATACCATATTGTTGATGGAAAAACATAAA
TTGATACGATATTGTTGATGGAAAAACGATACTATACTTAGTATCGATACTGAAAACCTTGAAAACTAGTACGGAGTGGTAAATAT
TTTCAGAACATTTTTTGTTCGAAGTAGAGGAAGAAGACAACTGGACCTGAGAATTCGTATACGACGGGCTATTTGCTGACACCGAC
ACGCGGGCCATCTCGTCTCTGAGCACGCAAGACGCAGCGCGGCTCCTTGGCTCGTTATCTCAGCGAGCACCGCAGCTCACACACGC
GTCGCCCCCAAGCTCGCTCTTCCCGA**ATG**

> PI231565-L
TTGGCTAACATAATCCCCTAACCTTCTACTAATCTGTTTTGCAGTCCCATTTTACCATGTTGAGACAAATTAGAAACCTACTTTTA
CATTGTAATCAGTGCTCGATCTGAATTTTCAATTAGTAAATATTTTAGTTTTAAAAACAATTATTTTCTATTATTTAAATTAGTTG
TCATAGATTTAGATAAAATTTCGTTTCATCAAACATGTAGAGAATTTGTCGAAATTAAACGTATCTAGACACTGAATAGTATAGAT

ATATTCAAACTTTAACAAATCTTAAACAACTTTTATGAAACGGAGGGAGCATTATCCATGTTCATTGCGACAACAAATTTGGATAA
AACTAGCATGGTTGTCCGCGATACATATCCTACTATCTTACAATACTAAGATCCTACTGGGTTGAAATGATATGTATCGTGTTGTG
TATCCTAATTAGGTGGTATCGTTGTTAGAATGTGATAACATGCCTTAAATCAGTAGTATCCCGATACTTCTACGACAGATGATACC
ACACATAAGTTAAAACTAATCAAATAATTAATTAACGTACAAACAAACAACAGTGTGACATCCTAGCCCAGGACTTAGTAGGATTG
ATAGGATACTCATATCAATAAGTTGTAACTTCTTTTCCGGAAATTCATCAGCAAACAACTCCGAGGTTAAGTGTGCTTGACCCGGA
GTAATTTGAGGATGGATGGCCGATCAGGAAGTAGTTTTCCTGTGTGCACGAGTGAGGACAAAATGCGCAGGAAAGACATGTGAGCT
GCGAGGGTAACATGCCCGGCCGGGGTGGTCGGGGTGATATATTTGGTATCAAAGCCGACCTTCGTGGTTACATGGGCATGTGCAGG
TCAGGGGTTCGGGTATGTGCACACGGCATGTCACATGTGCCAACACTGGATGCATAAACGTGTGCCAAGAAGGGTACGTTTCTGGC
CTAGAGCTAATCGACGAGTGTGTCGATCTTCTAAGGGGATGAGTGTGACATCCTAGCCCATGGCTTAATGCCAACAAGTTGTAACT
TCTTTTCCGGAAACTCATCAACAAAGAACTCCAAGGTTAAGCATGCTTGACCTTTTGTAATTTGAGGATGGGTAACCGATGGGGAA
GTGGTTTCCGAGGTGTGCACGAGTGAGAACAAAGTGCGCAGGAAAGACATGTGTTGGTCTATAAGGCTAGTCTAGAGATCATATAA
AGATCCTAGAGAGCTGCCGGCCGGTCCGACCGGGGTGATCGGGGTGTTACAAGGAGCGAGCCTTATAAGCAGGTAATAGCTTAACA
CATAAATTGATTTTATTTTGAAAAAAGTAGAAATCTCATAGTATCATTATACGATTCTATGATACCATATTGTTGATGGAAAAACA
TAAATTGATACGATATTGTTGATGGAAAAACGATACTATACTTAGTATCGATACGGAAAACCTTAAAAACTAGTACGGAGTAGTAA
ATATTTTCAGAACATTTTTTTGTTCGCAAGTAGAGGAAGAAGACAACTCGACCTGAGAATTCGTATACGACGGGCTATTTGCTGAC
ACCGACACGTGGGCCATCTCGTCTCTGAGCACGCAAGACGCAACGCGGCTCCTTGGCTCGTTATCTCAGCGAGCACCGCAGGCGCA
GCACACACACGCGTCGCCCCCAAGCTCGCTCTTCCCGA**ATG**

>PI182857-S
TTGGCTAACATAATCCCCTAACCTTCTACTAATCTGTTTTGCAGTCCCATTTTACCATGTTGAGACAAATTAGAAACCTACTTTTA
CATTGTAATCAGTGCTCGATCTGAATTTTCAATTAGTAAATATTTTAGTTTTAAAAACAATTATTTTCTATTATTTAAATTAGTTG
TCATAGATTTAGATAAAATTTCGTTTCATCAAACATGTAGAGAATTTGTCGAAATTAAACATATCTAGACACTGAATAGTATAGAT
ATATCCAAACTTTAACAAATCTTAAACAACTTTTATGAAACGGAGGGAGCATTATCCATGTTCATTGCGACAACAAATTTGGATAA
AACTAGCATGGTTGTCAGTATCGCGATACATATCCTATTGTCTTACAATACTAAGATCCTATTGGATTGAAATGATATGTATCGTG
TTGTGTATCCTAATTAGGTGGTATCGTCGTTAGAATACGATACCATGCCTTAAATCAGTAGTATCTCGATACTTCTACGTCAGATG
ATACCACACATAAGTTAAAACTAATTAAATAATAATTAATTAAATAGTATAGATATATCCCAGGTGACCGACCGGGAAGTGGTTTC
CAGGGTGCGCACGAGTGAGGAAAAACTGCGCAGGAAAGACATGTGTTGGTTCGTAGGGCTAGTCTAAAGATCCTATAGAGATCCTA
GAGAGCTGCCAGGGGTAACAGACCCGGCTGGGGTGGTCGGGGTGTTACAAGGAGCGAGCCTTATAAGCAGGTAATAGCTTAACACA
TAAATTGATTTTATTTTGAAAAGTACAAATCTCATAGTATCATTATACGATTCTATGATACCATATTGTTGATGGAAAAACATAAA
TTGATACGATATTGTTGATGGAAAAACGATACTATACTTAGTATCGATACTGAAAACCTTGAAAACTAGTACGGAGTGGTAAATAT
TTTCAGAACATTTTTTGTTCGAAGTAGAGGAAGAAGACAACTCGACCTGAGAATTCGTATACGACGGGCTATTTGCTGACACCGAC
ACGCGGGCCATCTCGTCTCTGAGCACGCAAGACGCAACGCGGCTCCTTTGGCTCGTTATCTCAGCGAGCACCGCAGGCGCGTCGCC
CCCACGCTCGCTCTTCCCGA**ATG**

> PI182857-L
TTGGCTAACATAATCCCCTAACCTTCTACTAATCTGTTTTGCAGTCCCATTTTACCATGTTGAGACAAATTAGAAACCTACTTTTA
CATTGTAATCAGTGCTCGATCTGAATTTTCAATTAGTAAATATTTTAGTTTTAAAAACAATTATTTTCTATTATTTAAATTAGTTG
TCATAGATTTAGATAAAATTTCGTTTCATCAAACATGTAGAGAATTTGTCGAAATTAAACGTATCTAGACACTGAATAGTATAGAT
ATATTCAAACTTTAACAAATCTTAAACAACTTTTATGAAACGGAGGGAGCATTATCCATGTTCATTGCGACAACAAATTTGGATAA
AACTAGCATGGTTGTCCGCGATACATATCCTACTATCTTACAATACTAAGATCCTACTGGGTTGAAATGATATGTATCGTGTTGTG
TATCCTAATTAGGTGGTATCGTTGTTAGAATGTGATAACATGCCTTAAATCAGTAGTATCCCGATACTTCTACGACAGATGATACC
ACACATAAGTTAAAACTAATCAAATAATTAATTAACGTACAAACAAACAACAGTGTGACATCCTAGCCCAGGACTTAGTAGGATTG
ATAGGATACTCATATCAATAAGTTGTAACTTCTTTTCCGGAAATTCATCAGCAAACAACTCCGAGGTTAAGTGTGCTTGACCCGGA
GTAATTTGAGGATGGATGGCCGATCAGGAAGTAGTTTTCCTGTGTGCACGAGTGAGGACAAAATGCGCAGGAAAGACATGTGAGCT
GCGAGGGTAACATGCCCGGCCGGGGTGGTCGGGGTGATATATTTGGTATCAAAGCCGACCTTCGTGGTTACATGGGCATGTGCAGG
TCAGGGGTTCGGGTATGTGCACACGGCATGTCACATGTGCCAACACTGGATGCATAAACGTGTGCCAAGAAGGGTACGTTTCTGGC
CTAGAGCTAATCGACGAGTGTGTCGATCTTCTAAGGGGATGAGTGTGACATCCTAGCCCATGGCTTAATGCCAACAAGTTGTAACT
TCTTTTCCGGAAACTCATCAACAAAGAACTCCAAGGTTAAGCATGCTTGACCTTTTGTAATTTGAGGATGGGTAACCGATGGGGAA
GTGGTTTCCGAGGTGTGCACGAGTGAGAACAAAGTGCGCAGGAAAGACATGTGTTGGTCTATAAGGCTAGTCTAGAGATCATATAA
AGATCCTAGAGAGCTGCCGGCCGGTCCGACCGGGGTGATCGGGGTGTTACAAGGAGCGAGCCTTATAAGCAGGTAATAGCTTAACA
CATAAATTGATTTTATTTTGAAAAAAGTAGAAATCTCATAGTATCATTATACGATTCTATGATACCATATTGTTGATGGAAAAACA
TAAATTGATACGATATTGTTGATGGAAAAACGATACTATACTTAGTATCGATACGGAAAACCTTAAAAACTAGTACGGAGTAGTAA
ATATTTTCAGAACATTTTTTTGTTCGCAAGTAGAGGAAGAAGACAACTCGACCTGAGAATTCGTATACGACGGGCTATTTGCTGAC
ACCGACACGTGGGCCATCTCGTCTCTGAGCACGCAAGACGCAACGCGGCTCCTTGGCTCGTTATCTCAGCGAGCACCGCAGGCGCA
GCACACACACGCGTCGCCCCCAAGCTCGCTCTTCCCGA**ATG**

>PI231604-S
TTGGCTAACATAATCCCCTAACCTTCTACTAATCTGTTTTGCAGTCCCATTTTACCATGTTGAGACAAATTAGAAACCTACTTTTA

CATTGTAATCAGTGCTCGATCTGAATTTTCAATTAGTAAATATTTTAGTTTTAAAAACAATTATTTTCTATTATTTAAATTAGTTG
TCATAGATTTAGATAAAATTTCGTTTCATCAAACATGTAGAGAATTTGTCGAAATTAAACATATCTAGACACTGAATAGTATAGAT
ATATCCAAACTTTAACAAATCTTAAACAACTTTTATGAAACGGAGGGAGCATTATCCATGTTCATTGCGACAACAAATTTGGATAA
AACTAGCATGGTTGTCAGTATCGCGATACATATCCTATTGTCTTACAATACTAAGATCCTATTGGATTGAAATGATATGTATCGTG
TTGTGTATCCTAATTAGGTGGTATCGTCGTTAGAATACGATACCATGCCTTAAATCAGTAGTATCTCGATACTTCTACGTCAGATG
ATACCACACATAAGTTAAAACTAATTAAATAATAATTAATTAAATAGTATAGATATATCCCAGGTGACCGACCGGGAAGTGGTTTC
CAGGGTGCGCACGAGTGAGGAAAAACTGCGCAGGAAAGACATGTGTTGGTTCGTAGGGCTAGTCTAAAGATCCTATAGAGATCCTA
GAGAGCTGCCAGGGGTAACAGACCCGGCTGGGGTGGTCGGGGTGTTACAAGGAGCGAGCCTTATAAGCAGGTAATAGCTTAACACA
TAAATTGATTTTATTTTGAAAAGTACAAATCTCATAGTATCATTATACGATTCTATGATACCATATTGTTGATGGAAAAACATAAA
TTGATACGATATTGTTGATGGAAAAACGATACTATACTTAGTATCGATACTGAAAACCTTGAAAACTAGTACGGAGTGGTAAATAT
TTTCAGAACATTTTTTGTTCGAAGTAGAGGAAGAAGACAACTCGACCTGAGAATTCGTATACGACGGGCTATTTGCTGACACCGAC
ACGCGGGCCATCTCGTCTCTGAGCACGCAAGACGCAACGCGGCTCCTTTGGCTCGTTATCTCAGCGAGCACCGCAGGCGCGTCGCC
CCCACGCTCGCTCTTCCCGA**ATG**

> PI231604-L
TTGGCTAACATAATCCCCTAACCTCCTACTAATCTGTTTTGCAGTCCCATTTTACCATGTTGAGACAAATTAGAAACCTACTTTTA
CATTGTAATCGGTGCTTGATCTGAATTTTCAATTAATAAATATTTTAGGTTTAAAAATAATTATTTTCTATTATTTAAATTAGTTG
TCATATATTTAGATAAAATATAGTAGTCCTTCCGTTTCATCAAGCATGTAGAGAATTTGTCAAAATTGAACGTATCTAGACACTGA
AAAGTATAGATATATCCGAATTTTAACAAATCTTAAACAACTTTTATGAAACGGAGGGAGCACTATCCATGTTCATTGCGACAACA
AATTTGGATAAAACTAGCATGGTTGTCAGTATCACGATACATATCCTACTATCTTACAGTACTAAGATCCTACTGGGTTGAAATGA
TATTTATCGTGTTGTGTATCCTAATTAGGTGGTATCGTTGTTAGAATACGATATCATGCCAAGTAGTATCCCGATACTTCTACGAC
AGATGATACCACACATAAGTTAAAACTAATCAAATAATTAATTAACGTACGAACAACAGTGTGACATCCTAGCCCAGAACTTAGTA
AGATTGATAGGATACTCATACCAATAAGTTGCAACTTCTTTTCTGGAAACTCATCAGCAAAGAACTCCGAGGTTAAGTGTTCTTGA
CCAGGAGTAATTTGAGGATGGATGACTGACCGGGAAGTAGTTCCTGTGTGCACGAGTGAGGACAAAATACGCAGGAAAGACATGTG
AGCTGCCAGGGGTAACATGCCCGGTCGGGGGTGGTCGGGGTGATACATTTGGTATCAAAGCCGACCTTCGCGGTTACCTGGGCATG
TGCGGGTCACAGGTTCGGGCATGTGCACACGGCATGTCACATGTGCCAGCACTGGATGCACAAACGTGTGCCAAGAAGGGGACGTT
TCTGGCCTGGGGCTAATCGACGAGGGCTTCGATCTTCTAAGGGGATGAGTGTGACATCCTAGCCCACAGCTTAATACCAACAAGTT
GCAATTTCTTTTCCGAAAACTCATCAACAAAGAACTCCGAGGTTAAGCGTGCTTGACCTATAATTTTTGAGGATGGGTGACCGATT
GAGAAGTGGTTTCCAGGGTGCGCACGAGTGATGATAAAGTGCGCATGAAAGACATGTGTTGATATGTAGATCTAGTCTAGAGATCA
TATAAAGATCCTAGAGAGCTGCTACAGGTAACAGGCCCAGGGTGGTCGGGGTGTTACAAGGAGCGAGCCTTATAAGCAGGTAATAG
CTTAACACATAAATTGATTTTATTTTGAAAAGTAGAAATCTCATAGTATCATGATACGATTCTATGATACCATATTGTTGGTGGAA
AAACATAAACTGATACGATATTGTTGATGGAAAAAACGATACTATACTTAGTATCGATACTGAAAACCTTAAAAACTAGTACGGGG
TAGTAAATATTTTCAGAACATATTTTGTTCGCAAGTACAGGAAGAAGACAAGTCGACCTGAGAATTCGTATACGACGGGCTATTTG
CTGACACCGACACGCGGGCCATCTCGTCTCTGAGCACGCAACGCAGCTCCTTGGCTCGTTATCTCAGCGAGCACCGCAGCTCACAC
ACGCGTCGCCCCCAAGCTCGCTCTTCCCGA**ATG**

>PI239730-S
TTGGCTAACATAATCCCCTAACCTCTCTAAGCCTAGATAAGTGCCCTGACCTCCTGCTAATCTGTTTTGCAGCCCAATTTTACCAT
GTTGAGACAAATTAGCTAGCAACCTACTTTTACATTGTTATCGGTGCTCTGAATTTTCACTTAATAAATCTTTTAGTTTTAAAAAT
AATTACTTCCTCTTATTCAAATTAGTTGTCACAGATTTAGATAAAATATAGTAGTCCCTCCGTTTCATCAAACATGTAGATAATTT
GTCGAAATTGAACTTATCTAGCCACCGGATAGTATAGATACATTCGAACTTGAACAAATCTTAAACAACTTTTATGAAACGGAGGG
AGCACTATCCTTGTTTATTACGACAACAAATTTGGATAAAACTAGCATGGTTGTCAGTACAGCTGGGCATGGGCAGCCCGGCCCGA
CCTGGTCCGAAAATCCCAACCCGGGCCGGGTCGGGCTTGCACTCAGGCCTGATATTTGAGCTCGAACGTTGGGTCGGGCTCAGGCA
TTCAATTTTGCGCGTTTTATCTGGCTCGGGCTGGGCCGGGCTTTTTGCTCATTTGGGCTAGGCACGTGCTAGCATTACAGGCAAAC
AATCGGGTCGGGCTGGGCTCGAGCCTGACTTTTTACCACTAGGCTTTTTTAGGCCCGGCCCGAAGCCTGGCACGGCCCAAACTGTG
TCCAGGTGTAGTTGTCAGTATCGCGATACAGATCCTAGCATCTTACGATACTAAGATCCTACCGGGGTCAAACGATACATATCATG
TTGAGTATCCTAATCAAGTGGTATCGTTGTGAGAATGCAATACCATGCCGTAAATTAGTAGTATCATGATACTTTTACAACAGATG
CTACCACACATAGGTTAAAACTAATCAAATAATTAGTTAACCTAGTGGCATCAGCAAGCCTTATAGCAGGTAATGGCTTCATTGGT
TTCCTACAGTGGACTAAACATACTATTTGCCACCTCTGCATTCCTTATGTCGTGTTGGACTGAATGCTCGAAATCTAGATTATGAT
ATAACATTCTCTTACGAGAATAATATGCATGACACTATGTTTTTATATAAATATATGGTAGATAACTATTTTATTTAACACATAAA
CTGATTATATTTTTAAAAGTAGAAATCTCATAGTGTCGTGATACTACAATATGATCCTATGGTACCATACTGTTGATGGAGAAACG
ATACTAATTAGTATCGATATATACTGAAAACCTTGAAAACTAGTAGTACGGAGTAGTAAATATTTTGAGAACATATTTTGTTCGCA
AGTACAGGAAGAAGACAACTCGGCCTGAGAATTCGTATACGACGGACTGCTTGCTTGCTGACACTGACACGCGGGCCATCTCGTCT
CTGAGCACGCAAGACGCACGCAACGCGGCTCCTTGGCTCGTTATCTCAGCGAGCACCGCAGGCGTCGCCCCCAAGCTCGCTCTTCC
CGA**ATG**

> PI239730-L
TTGGCTAACATAATCCCCTAACCTTCTACTAATCTGTTTTGCAGTCCCATTTTACCATGTTGAGACAAATTAGAAACCTACTTTTA

CATTGTAATCAGTGCTCGATCTGAATTTTCAATTAGTAAATATTTTAGTTTTAAAAACAATTATTTTCTATTATTTAAATTAGTTG
TCATAGATTTAGATAAAATTTCGTTTCATCAAACATGTAGAGAATTTGTCGAAATTAAACGTATCTAGACACTGAATAGTATAGAT
ATATTCAAACTTTAACAAATCTTAAACAACTTTTATGAAACGGAGGGAGCATTATCCATGTTCATTGCGACAACAAATTTGGATAA
AACTAGCATGGTTGTCCGCGATACATATCCTACTATCTTACAATACTAAGATCCTACTGGGTTGAAATGATATGTATCGTGTTGTG
TATCCTAATTAGGTGGTATCGTTGTTAGAATGTGATAACATGCCTTAAATCAGTAGTATCCCGATACTTCTACGACAGATGATACC
ACACATAAGTTAAAACTAATCAAATAATTAATTAACGTACAAACAAACAACAGTGTGACATCCTAGCCCAGGACTTAGTAGGATTG
ATAGGATACTCATATCAATAAGTTGTAACTTCTTTTCCGGAAATTCATCAGCAAACAACTCCGAGGTTAAGTGTGCTTGACCCGGA
GTAATTTGAGGATGGATGGCCGATCAGGAAGTAGTTTTCCTGTGTGCACGAGTGAGGACAAAATGCGCAGGAAAGACATGTGAGCT
GCGAGGGTAACATGCCCGGCCGGGGTGGTCGGGGTGATATATTTGGTATCAAAGCCGACCTTCGTGGTTACATGGGCATGTGCAGG
TCAGGGGTTCGGGTATGTGCACACGGCATGTCACATGTGCCAACACTGGATGCATAAACGTGTGCCAAGAAGGGTACGTTTCTGGC
CTAGAGCTAATCGACGAGTGTGTCGATCTTCTAAGGGGATGAGTGTGACATCCTAGCCCATGGCTTAATGCCAACAAGTTGTAACT
TCTTTTCCGGAAACTCATCAACAAAGAACTCCAAGGTTAAGCATGCTTGACCTTTTGTAATTTGAGGATGGGTAACCGATGGGGAA
GTGGTTTCCGAGGTGTGCACGAGTGAGAACAAAGTGCGCAGGAAAGACATGTGTTGGTCTATAAGGCTAGTCTAGAGATCATATAA
AGATCCTAGAGAGCTGCCGGCCGGTCCGACCGGGGTGATCGGGGTGTTACAAGGAGCGAGCCTTATAAGCAGGTAATAGCTTAACA
CATAAATTGATTTTATTTTGAAAAAAGTAGAAATCTCATAGTATCATTATACGATTCTATGATACCATATTGTTGATGGAAAAACA
TAAATTGATACGATATTGTTGATGGAAAAACGATACTATACTTAGTATCGATACGGAAAACCTTAAAAACTAGTACGGAGTAGTAA
ATATTTTCAGAACATTTTTTTGTTCGCAAGTAGAGGAAGAAGACAACTCGACCTGAGAATTCGTATACGACGGGCTATTTGCTGAC
ACCGACACGTGGGCCATCTCGTCTCTGAGCACGCAAGACGCAACGCGGCTCCTTGGCTCGTTATCTCAGCGAGCACCGCAGGCGCA
GCACACACACGCGTCGCCCCCAAGCTCGCTCTTCCCGA**ATG**

>PI610950-S
TTGGCTAACATAATCCCCTAACCTTCTACTAATCTGTTTTGCAGTCCCATTTTACCATGTTGAGACAAATTAGAAACCTACTTTTA
CATTGTAATCAGTGCTCGATCTGAATTTTCAATTAGTAAATATTTTAGTTTTAACAACAATTATTTTCTATTATTTAAATTAGTTG
TCATAGATTTAGATAAAATTTCGTTTCATCAAACATGTAGAGAATTTGTCGAAATTAAACATATCTAGACACTGAATAGTATAGAT
ATATCCAAACTTTAACAAATCTTAAACAACTTTTATGAAACGGAGGGAGCATTATCCATGTTCATTGCGACAACAAATTTGGATAA
AACTAGCATGGTTGTCAGTATCGCGATACATATCCTATTGTCTTACAATACTAAGATCCTATTGGGTTGAAATGATATGTATCGTG
TTGCGTATCCTAATTAGGTGGTATCGTCGTTAGAATACGATACCATGCCTTAAATCAGTAGTATCTCGATACTTCTACGTCAGATG
ATACCACACATAAGTTAAAACTAATTAAATAATAATTAATTAAATAGTATAGATATATCCCAGGTGACAGACCGGAAAGTGGTTTT
CAGGGTACGCACGAGTGAGGAAAAACTGCGCAGGAAAGACATGTGTTGATTCGTAGGGCTAGTCTAGAGATCCTATAGAGATCCTA
GAGAGCTGCCAGGGGTAACAGACCCGGCTGGGCTGGTCGGGGTGTTACAAGGAGCGAGCCTTATAAGCAGGTAATAGCTTAACACA
TAAATTGATTTTATTTTGAAAAGTAGAAATCTCATAGTATCATTATACGATTCTATGATACCATATTTTGTTGATGGAAAAACATA
AATTGATACGATATTGCTGATGGAAAAACGATACTATAATTAGTATCGATACTGAAAACCTTGAAACTGTACGGAGTGGTAAATAT
TTTCAGAACATTTTTTGTTCGCAAGTAGAGGAAGAAGACAACTGGACCTGAGAATTCGTATACGACGGGCTATTTGCTGACACCGA
CACGCGGGCCATCTCGTCTCTGAGCACGCAACGCAGCTCCTTGGCTCGTTATCTCAGCGACAGCGAGCACCGCAGCGCGCACACGC
GTCGCCCCCAAGCTCGCTCTTCCCGA**ATG**

> PI610950-L
TTGGCTAAGATAATCCCCTAACCTTCTACTAATCTGTTTTGCAGTCCCATTTTACCATGTTGAGACAAATTAGAAACCTACTTTTA
CATTGTAATCAGTGCTCGATCTGAATTTTCAATTAGTAAATATTTTAGTTTTAAAAACAATTATTTTCTATTATTTAAATTAGTTG
TCATAGATTTAGATAAAATTTCGTTTCATCAAACATGTAGAGAATTTGTCGAAATTAAACGTATCTAGACACTGAATAGTATAGAT
ATATTCAAACTTTAACAAATCTTAAACAACTTTTATGAAACGGAGGGAGCATTATCCATGTTCATTGCGACAACAAATTTGGATAA
AACTAGCATGGTTGTCCGCGATACATATCCTACTATCTTACAATACTAAGATCCTACTGGGTTGAAATGATATGTATCGTGTTGTG
TATCCTAATTAGGTGGTATCGTTGTTAGAATGTGATAACATGCCTTAAATCAGTAGTATCCCGATACTTCTACGACAGATGATACC
ACACATAAGTTAAAACTAATCAAATAATTAATTAACGTACAAACAAACAACAGTGTGACATCCTAGCCCAGGACTTAGTAGGATTG
ATAGGATACTCATATCAATAAGTTGTAACTTCTTTTCCGGAAATTCATCAGCAAACAACTCCGAGGTTAAGTGTGCTTGACCCGGA
GTAATTTGAGGATGGATGGCCGATCAGGAAGTAGTTTTCCTGTGTGCACGAGTGAGGACAAAATGCGCAGGAAAGACATGTGAGCT
GCGAGGGTAACATGCCCGGCCGGGGTGGTCGGGGTGATATATTTGGTATCAAAGCCGACCTTCGTGGTTACATGGGCATGTGCAGG
TCAGGGGTTCGGGTATGTGCACACGGCATGTCACATGTGCCAACACTGGATGCATAAACGTGTGCCAAGAAGGGTACGTTTCTGGC
CTAGAGCTAATCGACGAGTGTGTCGATCTTCTAAGGGGATGAGTGTGACATCCTAGCCCATGGCTTAATGCCAACAAGTTGTAACT
TCTTTTCCGGAAACTCATCAACAAAGAACTCCAAGGTTAAGCATGCTTGACCTTTTGTAATTTGAGGATGGGTAACCGATGGGGAA
GTGGTTTCCGAGGTGTGCACGAGTGAGAACAAAGTGCGCAGGAAAGACATGTGTTGGTCTATAAGGCTAGTCTAGAGATCATATAA
AGATCCTAGAGAGCTGCCGGCCGGTCCGACCGGGGTGATCGGGGTGTTACAAGGAGCGAGCCTTATAAGCAGGTAATAGCTTAACA
CATAAATTGATTTTATTTTGAAAAAAGTAGAAATCTCATAGTATCATTATACGATTCTATGATACCATATTGTTGATGGAAAAACA
TAAATTGATACGATATTGTTGATGGAAAAACGATACTATACTTAGTATCGATACGGAAAACCTTAAAAACTAGTACGGAGTAGTAA
ATATTTTCAGAACATTTTTTTGTTCGCAAGTAGAGGAAGAAGACAACTCGACCTGAGAATTCGTATACGACGGGCTATTTGCTGAC
ACCGACACGTGGGCCATCTCGTCTCTGAGCACGCAAGACGCAACGCGGCTCCTTGGCTCGTTATCTCAGCGAGCACCGCAGGCGCA
GCACACACACGCGTCGCCCCCAAGCTCGCTCTTCCCGA**ATG**

>PI577251-S
TTGGCTAACATAATCCCCTAACCTTCTACTAATCTGTTTTGCAGTCCCATTTTACCATGTTGAGACAAATTAGAAACCTACTTTTA
CATTGTAATCAGTGCTCGATCTGAATTTTCAATTAGTAAATATTTTAGTTTTAAAAACAATTATTTTCTATTATTTAAATTAGTTG
TCATAGATTTAGATAAAATTTCGTTTCATCAAACATGTAGAGAATTTGTCGAAATTAAACATATCTAGACACTGAATAGTATAGAT
ATATCCAAACTTTAACAAATCTTAAACAACTTTTATGAAACGGAGGGAGCATTATCCATGTTCATTGCGACAACAAATTTGGATAA
AACTAGCATGGTTGTCAGTATCGCGATACATATCCTATTGTCTTACAATACTAAGATCCTATTGGATTGAAATGATATGTATCGTG
TTGTGTATCCTAATTAGGTGGTATCGTCGTTAGAATACGATACCATGCCTTAAATCAGTAGTATCTCGATACTTCTACGTCAGATG
ATACCACACATAAGTTAAAACTAATTAAATAATAATTAATTAAATAGTATAGATATATCCCAGGTGACCGACCGGGAAGTGGTTTC
CAGGGTGCGCACGAGTGAGGAAAAACTGCGCAGGAAAGACATGTGTTGGTTCGTAGGGCTAGTCTAAAGATCCTATAGAGATCCTA
GAGAGCTGCCAGGGGTAACAGACCCGGCTGGGGTGGTCGGGGTGTTACAAGGAGCGAGCCTTATAAGCAGGTAATAGCTTAACACA
TAAATTGATTTTATTTTGAAAAGTACAAATCTCATAGTATCATTATACGATTCTATGATACCATATTGTTGATGGAAAAACATAAA
TTGATACGATATTGTTGATGGAAAAACGATACTATACTTAGTATCGATACTGAAAACCTTGAAAACTAGTACGGAGTGGTAAATAT
TTTCAGAACATTTTTTGTTCGAAGTAGAGGAAGAAGACAACTCGACCTGAGAATTCGTATACGACGGGCTATTTGCTGACACCGAC
ACGCGGGCCATCTCGTCTCTGAGCACGCAAGACGCAACGCGGCTCCTTTGGCTCGTTATCTCAGCGAGCACCGCAGGCGCGTCGCC
CCCACGCTCGCTCTTCCCGA**ATG**

> PI577251-L
TTGGCTAACATAATCCCCTAACCTTCTACTAATCTGTTTTGCAGTCCCATTTTACCATGTTGAGACAAATTAGAAACCTACTTTTA
CATTGTAATCAGTGCTCGATCTGAATTTTCAATTAGTAAATATTTTAGTTTTAAAAACAATTATTTTCTATTATTTAAATTAGTTG
TCATAGATTTAGATAAAATTTCGTTTCATCAAACATGTAGAGAATTTGTCGAAATTAAACGTATCTAGACACTGAATAGTATAGAT
ATATTCAAACTTTAACAAATCTTAAACAACTTTTATGAAACGGAGGGAGCATTATCCATGTTCATTGCGACAACAAATTTGGATAA
AACTAGCATGGTTGTCCGCGATACATATCCTACTATCTTACAATACTAAGATCCTACTGGGTTGAAATGATATGTATCGTGTTGTG
TATCCTAATTAGGTGGTATCGTTGTTAGAATGTGATAACATGCCTTAAATCAGTAGTATCCCGATACTTCTACGACAGATGATACC
ACACATAAGTTAAAACTAATCAAATAATTAATTAACGTACAAACAAACAACAGTGTGACATCCTAGCCCAGGACTTAGTAGGATTG
ATAGGATACTCATATCAATAAGTTGTAACTTCTTTTCCGGAAATTCATCAGCAAACAACTCCGAGGTTAAGTGTGCTTGACCCGGA
GTAATTTGAGGATGGATGGCCGATCAGGAAGTAGTTTTCCTGTGTGCACGAGTGAGGACAAAATGCGCAGGAAAGACATGTGAGCT
GCGAGGGTAACATGCCCGGCCGGGGTGGTCGGGGTGATATATTTGGTATCAAAGCCGACCTTCGTGGTTACATGGGCATGTGCAGG
TCAGGGGTTCGGGTATGTGCACACGGCATGTCACATGTGCCAACACTGGATGCATAAACGTGTGCCAAGAAGGGTACGTTTCTGGC
CTAGAGCTAATCGACGAGTGTGTCGATCTTCTAAGGGGATGAGTGTGACATCCTAGCCCATGGCTTAATGCCAACAAGTTGTAACT
TCTTTTCCGGAAACTCATCAACAAAGAACTCCAAGGTTAAGCATGCTTGACCTTTTGTAATTTGAGGATGGGTAACCGATGGGGAA
GTGGTTTCCGAGGTGTGCACGAGTGAGAACAAAGTGCGCAGGAAAGACATGTGTTGGTCTATAAGGCTAGTCTAGAGATCATATAA
AGATCCTAGAGAGCTGCCGGCCGGTCCGACCGGGGTGATCGGGGTGTTACAAGGAGCGAGCCTTATAAGCAGGTAATAGCTTAACA
CATAAATTGATTTTATTTTGAAAAAAGTAGAAATCTCATAGTATCATTATACGATTCTATGATACCATATTGTTGATGGAAAAACA
TAAATTGATACGATATTGTTGATGGAAAAACGATACTATACTTAGTATCGATACGGAAAACCTTAAAAACTAGTACGGAGTAGTAA
ATATTTTCAGAACATTTTTTTGTTCGCAAGTAGAGGAAGAAGACAACTCGACCTGAGAATTCGTATACGACGGGCTATTTGCTGAC
ACCGACACGTGGGCCATCTCGTCTCTGAGCACGCAAGACGCAACGCGGCTCCTTGGCTCGTTATCTCAGCGAGCACCGCAGGCGCA
GCACACACACGCGTCGCCCCCAAGCTCGCTCTTCCCGA**ATG**

>PI231567-S
TTGGCTAACATAATCCCCTAACCTTCTACTAATCTGTTTTGCAGTCCCATTTTACCATGTTGAGACAAATTAGAAACCTACTTTTA
CATTGTAATCAGTGCTCGATCTGAATTTTCAATTAGTAAATATTTTAGTTTTAAAAACAATTATTTTCTATTATTTAAATTAGTTG
TCATAGATTTAGATAAAATTTCGTTTCATCAAACATGTAGAGAATTTGTCGAAATTAAACATATCTAGACACTGAATAGTATAGAT
ATATCCAAACTTTAACAAATCTTAAACAACTTTTATGAAACGGAGGGAGCATTATCCATGTTCATTGCGACAACAAATTTGGATAA
AACTAGCATGGTTGTCAGTATCGCGATACATATCCTATTGTCTTACAATACTAAGATCCTATTGGGTTGAAATGATATGTATCGTG
TTGTGTATCCTAATTAGGTGGTATCGTCGTTAGAATACGATACCATGCCTTAAATCAGTAGTATCTCGATACTTCTACGTCAGATG
ATACCACACATAAGTTAAAACTAATTAAATAATAATTAATTAAATAGTATAGATATATCCCAGGTGACCGACCGGGAAGTGGTTTC
CAGGGTGCGCACGAGTGAGGAAAAACTGCGTAGGAAAGACATGTGTTGGTTCGTAGGGCTAGTCTAGAGATCCTATAGAGATCCTA
GAGAGCTGCCAGGGGTAACAGACCCGGCTGGGGTGGTCGGGGTGTTACAAGGAGCGAGCCTTATAAGCAGGTAATAGCTTAACACA
TAAATTGATTTTATTTTGAAAAGTAGAAATCTCATAGTATCATTATACGATTCTATGATACCATATTGTTGATGGAAAAACATAAA
TTGATACGATATTGTTGATGGAAAAACGATACTATACTTAGTATCGATACTGAAAACCTTGAAAACTAGTACGGAGTGGTAAATAT
TTTCAGAACATTTTTTGTTCGAAGTAGAGGAAGAAGACAACTGGACCTGAGAATTCGTATACGACGGGCTATTTGCTGACACCGAC
ACGCGGGCCATCTCGTCTCTGAGCACGCAAGACGCAGCGCGGCTCCTTGGCTCGTTATCTCAGCGAGCACCGCAGCTCACACACGC
GTCGCCCCCAAGCTCGCTCTTCCCGA**ATG**

> PI231567-L
TTGGCTAACATAATCCCCTAACCTTCTACTAATCTGTTTTGCAGTCCCATTTTACCATGTTGAGACAAATTAGAAACCTACTTTTA
CATTGTAATCAGTGCTGATCTGAATTTTCAATTAGTAAGTTTTAAAAATAATTATTTTCTATTATTTAAATTAGTTGTCATAGATT
TAGATAAAATTCGGTTTCATCAAACATGTAGAGAATTTGTCGAAATTGAACGTATCTAGACACTGAATAGTATAGATATATCCGAA

CTTTAACAAATCTTAAACAACTTTTATGAAACGGAGGGAGCATTATCCATGTTCATTGCGACAACAAATTTGGATAAAAGTAGCAT
GGTCGTCAGTACCGCGATACATATCATATTATCTTACAATACTAAGATCCTACTTGGTTGAAATGATATGTATCGTGTTGTGTATC
CTAATTAGGTGGTATCGTTGTTAGAATGCGATACCATGCCTTAAATCAGTAGTATCCCGATACTTCTACGACAGATGAAACCACAC
ATAAGCTAAAATTAATCAAATAATTAATTAACCTACGAACGGCAGGGTGACATCCTAGCCCAGGACTTAGTAGGAGTGATAGGATA
CCCATATCAACAAGTTGCAACTTCTTTTCCGAAAACTCATCAGCAAAGAACTTTGAGGTTAAGTGTGCTTGATTCGGAGTAATTTG
AGAATGGATGACCGACCGAGAAGTAGTTTCCCTGTGTGCACGAGTGAGGACAAAATGCGCAGGAAAGATATGTGAGTTGCGAGGGG
TAACATGCCCAGCCGGGGGTGGTCGGGGTGATACATTTGGTATCAAAACCGACCTTCACGGTTACATGGGCATGTGCAGGTCAGGG
GTTCGGGCATGTGCACACGGCATGTCACATGTGCCAACACTGGATGCATAAACGTGTGCCAAGAAGGGTACGTTTCTGGCCTGGGG
CTAATCGACGAGGGCATCGATCTTCTAAGGAGATGAGTGTGACATCCTAGCCCACGGCTTAATACCAACAAGTTACAATTTCTTTT
CCGAAAACTCATCAACAAAGAACTCCGAGGTTAAGCGTGCTTGACCTATAGTATTTGAGGATGGGTGACTGACCGGAAGTGGTTTC
CGGAGTGCGCACGAGTGAGTACAAAATACGCAAGAAAGATATGTGTTGGTATGTGGAGCTAGTCTAGAGATCATATAAAGATCCTA
GAGAGCTGCCACAGGTAACAGGCCCGGCCGGGGTGGTCGGGGTGTTACAAGGTGCGATCCTATAAGCAGGTAATAGCTTAACACAT
AAATTGATTTTATTTTGAAAAGTAGAAATCTCATAGTATCATGATACGATTCTATGATACGATATTGCTGATGAAAAACGATACTC
CCTCCGGTTCATATTAATTGACTCTAATATGGATGTATCTAGAACTGAAATGTGTCTAGATACATCCATATTGAAGTCAATTAATA
TGAATCGGAGGGAGTACTATACTTAGTATCGATACGGAATACCTTGAAAACTAGTACGGAGTAGTAAATATTTTCAGAACATTTTT
TGTTCGCGAGTACAGGAAGAAGACAACTGGACCTGAGAATTCGTATACGATGGGCTATTTGCTGACACCGACACGCGGGCCATCTC
GTCTCTGAGCACGCAAGACGCACGCAACGCAGCTCCTTGGCTCGTTATCTCAGCGAGCACCGCAGCGCGCACACGCGTCGCCCCCA
AGCTCGCTCTTCCCGA**ATG**

>PI303027-S
TTGGCTAACATAATCCCCTAACCTTCTACTAATCTGTTTTGCAGTCCCATTTTACCATGTTGAGACAAATTAGAAACCTACTTTTA
CATTGTAATCAGTGCTCGATCTGAATTTTCAATTAGTAAATATTTTAGTTTTAAAAACAATTATTTTCTATTATTTAAATTAGTTG
TCATAGATTTAGATAAAATTTCGTTTCATCAAACATGTAGAGAATTTGTCGAAATTAAACATATCTAGACACTGAATAGTATAGAT
ATATCCAAACTTTAACAAATCTTAAACAACTTTTACGAAACGGAGGGAGCATTATCCATGTTCATTGCGACAACAAATTTGGATAA
AACTAGCATGGTTGTCAGTATCGCGATACATATCCTATTGTCTTACAATACTAAGATCCTATTGGGTTGAAATGATATGTATCGTG
TTGTGTATCCTAATTAGGTGGTATCGTCGTTAGAATACGATACCATGCCTTAAATCAGTAGTATCTCGATACTTCTACGTCAGATG
ATACCACACATAAGTTAAAACTAATTAAATAATAATTAATTAAATAGTATAGATATATCCCAGGTGACCGACCGGGAAGTGGTTTC
CAGGGTGCGCACGAGTGAGGAAAAACTGCGCAGGAAAGACATGTGTTGGTTCGTAGGGCTAGTCTAGAGATCCTATAGAGATCCTA
GAGAGCTGCCAGGGGTAACAGACCCGGCTGGGGTGGTCGGGGTGTTACAAGGAGCGAGCCTTATAAGCAGGTAATAGCTTAACACA
TAAATTGATTTTATTTTGAAAAGTAGAAATCTCATAGTATCATTATACGATTCTATGATACCATATTGTTGATGGAAAAACATAAA
TTGATACGATATTGTTGATGGAAAAACGATACTATACTTAGTATCGATACTGAAAACCTTGAAAACTAGTACGGAGTGGTAAATAT
TTTCAGAACATTTTTTGTTCGAAGTAGAGGAAGAAGACAACTCGACCTGAGAATTCGTATACGACGGGCTATTTGCTGACACCGAC
GCGCGGGCCATCTCGTCTCTGAGCACGCAAGACGCAACGCGGCTCCTTTGGCTCGTTATCTCAGCGAGCACCGCAGGCGCGTCGCC
CCCACGCTCGCTCTTCCCGA**ATG**

> PI303027-L
TTGGCTAACATAATCCCCTAACCTTCTACTAATCTGTTTTGCAGTCCCATTTTACCATGTTGAGACAAATTAGAAACCTACTTTTA
CATTGTAATCAGTGCTCGATCTGAATTTTCAATTAGTAAATATTTTAGTTTTAAAAATAATTATTTTCTATTATTTAAATTAGTTG
TCATAGATTTAGATAAAATTTCGTTTCATCAAACATGTAGAGAATTTGTCGAAATTAAACGTATCTAGACACTGAATAGTATAGAT
ATATCCAAACTTTAACAAATCTTAAACAACTTTTATGAAACGGAGGGAGCATTATCCATGTTCATTGCGACAACAAATTTGGATAA
AACTAGCATGGTTGTCAGTATCGCGATACATATCCTACTATCTTACAATACTAAGATCCTACTGGGTTGAAATGATATGCATCGTG
TTGTGTATCCTAATTAGGTGGTATCGTTGTTAGAATGTGATACAATGCCTTAAATCAGTAGTATCCCGATACTTCTACGACATATG
ATAATACCACACATAAGTTAAAACTAATCAAATAATTAATTAACGTACAAACAACAGTGTGACATCCTAGCCCAGGATTTAGTAGG
ATTGATAGGATACTCATATTAATAAGTTGTAACTTCTTTTCCGAAAACTCATCAGCAAAGAACTCCGAGGTTAAGTGTGCTTGACC
CGGAGTAATTTGAGGATGGATGACCGACCGGGAAGTAGTTTCCCTGTGTGCACGAGTGAGAACAAAATACGCAGGAAAGACATGTG
AGCTGCCAGGGATAACATGCCTGGCTGGAGGTGGTCGGGGTGATACATTTGGTATCAAAGCCGACCTTCGCGGTTACATGGACATG
TGCAGGTCAGGGGTTCAGGCATGTGCACACGGCATGTCACATGTGCCAACACTAGATGCACAAACGTGTGCCAAGAAGGGTACGTT
TCTGGCCTAGAGCTAATCGACGAGGGCGTCGATCTTCTAAGGGGATGAGTGTGACATCCTAGCCCATGGCTTAATACCAACAAGTT
GCAACTTCTTTTCCGGAAACTCATCAACAAAGAACTCGAGGTTAAGCATGCTTGACCTATAGTATTTGAGGATGAGTGACCGATCG
GGAAGTGGTTTTCGAGGTGTGCACGAGTGAGAACAAAATGCGCAGGAAAGACATGTGTTGGTCTATGAGGCTAGTCTAGAGATCAT
ATAAAGATCCTAGAGAGCTGCCACAGATAACGGGCCGGTCCGACCAGGGTGATCGGGGTGTTACAAGGAGCGAGCCTTATAAGCAG
GTAATAGCTTAACACATAAATTGATTTTATTTTGAAAAGTAGAAAATCTCATAGTATCATTATACGATTCTATGATACCATATTTT
GTTGATGGAAAAAACATAAATTGATACGATATTGTTGATGGAAAAACGATACTATACTTAGTATCGATACGGAAAACCTTGAAATT
TAGTACGGAGTAGTAAATATTTTCAGAACATTTTTTGTTCGCAAGTAGAGGAAGAAGACAACTCGACCTGAGAATTCGTATACGAC
GGGCTATTTGCTGACACCGACACGCGGGCCATCTCGTCTCTGAGCACGCAACGCAGCTCCTTGGCTCGTTATCTCAGCGACAGCGA
GCACCGCAGCGCGCACACGCGTCGCCCCCAAGCTCGCTCTTCCCGA**ATG**
